# Docosahexaenoic acid mechanisms of action on the bovine oocyte-cumulus complex

**DOI:** 10.1186/s13048-017-0370-z

**Published:** 2017-11-09

**Authors:** Sebastien Elis, Mouhamad Oseikria, Anais Vitorino Carvalho, Priscila Silvana Bertevello, Emilie Corbin, Ana-Paula Teixeira-Gomes, Jérôme Lecardonnel, Catherine Archilla, Véronique Duranthon, Valérie Labas, Svetlana Uzbekova

**Affiliations:** 1 UMR PRC, CNRS, IFCE, INRA, Université de Tours, 37380 Nouzilly, France; 2 UMR BDR, ENVA, INRA, Université Paris-Saclay, 78350 Jouy-en-Josas, France; 3 UMR ISP, INRA, Université de Tours, 37380 Nouzilly, France; 4 INRA, Plateforme d’Analyse Intégrative des Biomolécules, Laboratoire de Spectrométrie de Masse, 37380 Nouzilly, France; 5 GABI, INRA, Agroparis Tech, Université de Paris-Saclay, 78350 Jouy-en-Josas, France

**Keywords:** Bovine, Oocyte-cumulus complex, FFAR4, DHA, Oocyte maturation

## Abstract

**Background:**

Supplementation of bovine oocyte-cumulus complexes during in vitro maturation (IVM) with 1 μM of docosahexaenoic acid (DHA), C22:6 n-3 polyunsaturated fatty acid, was reported to improve in vitro embryo development. The objective of this paper was to decipher the mechanisms of DHA action.

**Results:**

Transcriptomic analysis of 1 μM DHA-treated and control cumulus cells after 4 h IVM showed no significant difference in gene expression. MALDI-TOF mass spectrometry analysis of lipid profiles in DHA-treated and control oocytes and cumulus cells after IVM showed variations of only 3 out of 700 molecular species in oocytes and 7 out of 698 species in cumulus cells (*p* < 0.01).

We showed expression of free fatty acid receptor *FFAR4* in both oocytes and cumulus cells, this receptor is known to be activated by binding to DHA. FFAR4 protein was localized close to the cellular membrane by immunofluorescence. Functional studies demonstrated that supplementation with FFAR4 agonist TUG-891 (1 μM or 5 μM) during IVM led to an increased blastocyst rate (39.5% ± 4.1%, 41.3% ± 4.1%), similar to DHA 1 μM treatment (39.2% ± 4.1%) as compared to control (25.2% ± 3.6%).

FFAR4 activation via TUG-891 led to beneficial effect on oocyte developmental competence and might explain in part similar effects of DHA.

**Conclusions:**

In conclusion, we suggested that low dose of DHA (1 μM) during IVM might activate regulatory mechanisms without evident effect on gene expression and lipid content in oocyte-cumulus complexes, likely through signaling pathways which need to be elucidated in further studies.

**Electronic supplementary material:**

The online version of this article (10.1186/s13048-017-0370-z) contains supplementary material, which is available to authorized users.

## Background

Polyunsaturated fatty acids (PUFA) belong to a family of biologically active fatty acids (FA) known to have numerous health benefits [1]. Among them, n-3 PUFA, α-linolenic acid (ALA, C18:3 n-3), eicosapentaenoic acid (EPA, C20:5 n-3) and docosahexaenoic acid (DHA, C22:6 n-3) are essential FA, especially because mammals cannot produce ALA, which is the precursor of EPA and DHA. DHA, the longest member of this family, can be produced from ALA and EPA; however, the efficiency of the necessary elongation and desaturation reactions in mammals is low, and the most extensive source of DHA is therefore the diet, particularly seafood [2, 3]. In several organisms, DHA has been described as exerting pleiotropic effects at both the central and peripheral levels (i.e. brain, heart, inflammation) [4–6].

Among their physiological roles, n-3 PUFA affect reproduction in cattle [7, 8]. Indeed, an n-3 PUFA-enriched diet led to a significant increase or a tendency toward a higher presumptive conception rate [9–11] or reduced early embryo loss in cows [8–10, 12]. Moreover, an n-3 PUFA diet also directly affected the cleavage rate and tended to increase the blastocyst rate after in vitro maturation (IVM) and in vitro fertilization (IVF) in cows [13, 14]. We also reported in a previous study that DHA supplementation during IVM was able to increase the blastocyst rate of bovine oocytes after IVF, and that this beneficial effect was only observed with a low physiological dose of 1 μM [15]. Regarding the possible mechanism of the DHA effect, we also measured in that study the expression of several candidate genes involved in lipid metabolism, but did not manage to report any significant differences at the low dose of 1 μM DHA [15].

There are several possible mechanisms by which n-3 PUFA can exert multiple physiological roles in organism development and diseases [16]. These mechanisms include indirect actions on cells by influencing concentrations of metabolites and hormones, or the oxidation of LDL and oxidative stress, and direct actions on cells via receptors, sensors or cell membrane fatty acid composition (membrane order, lipid rafts, etc.) (reviewed in [17]). Most of the studies reporting n-3 PUFA mechanisms concerned the inflammation process [18]. Indeed, n-3 PUFA have been shown to affect cytokine expression, especially inflammatory cytokines (TNF, IL-1β, IL-6 and IL-8) and at the same time to help produce inflammatory resolving metabolites (resolvins, protectins, maresins) [17, 19]. Moreover, with an n-3 PUFA enriched diet, the membrane phospholipid composition was increased (reviewed in [19]). The production of eicosanoids (prostaglandins, leukotrienes, thromboxanes, lipoxins) from n-3 PUFA (i.e. series 3 prostaglandins) was therefore increased and, conversely, eicosanoid production from arachidonic acid (i.e. series 2 prostaglandins) was reduced. Series 3 prostaglandins have a lower affinity for their receptors and therefore their inflammatory effect is reduced. N-3 PUFA have also been reported to act through intracellular sensors, such as transcription factors, i.e. the peroxisome proliferator activated receptor (PPAR) and nuclear factor kappa B (NFκB) and thus modulate target gene expression [17]. By affecting the membrane lipid composition, n-3 PUFA were also able to increase membrane fluidity and to promote lipid raft formation, thereby enhancing signaling pathways. N-3 PUFA have also been reported to act through membrane free fatty acid receptors, such as FFAR1 and FFAR4, and activate their signaling pathways [20]. Indeed, FFAR4 can activate mitogen activated protein kinase MAPK1/3 (alias ERK 1/2) signaling [21] which is already known to be activated in mouse cumulus cells during oocyte maturation [22] as well as in bovine oocytes [23]. A significant increase of MAPK1/3 phosphorylation was also reported in bovine cumulus cells (CC) after IVM compared to in vivo [24].

In this study, we hypothesized that the mechanisms of action of DHA, associated with the previously reported effects of DHA on oocyte quality, might occur through either modulation of gene expression and/or lipid composition and/or fatty acid receptor activation. The first objective was thus to highlight the mechanisms of action of DHA on oocyte-cumulus complex (OCC) by analyzing whether gene expression, lipid composition, or MAPK3/1 signaling were affected by DHA treatment. The second objective was to test whether stimulation of the FFAR4 receptor in the OCC during IVM could be sufficient to reproduce the effects of DHA on oocyte quality. To achieve this objective, we first analyzed FFAR4 expression in OCC and then analyzed whether supplementation with an FFAR4 agonist, TUG-891 [25], would reproduce the beneficial effect of DHA previously reported on bovine oocyte competence and in vitro embryo development [15].

## Methods

### Ethics

No experiments with living animals were performed.

### Chemicals and antibody

All chemicals were obtained from Sigma-Aldrich (Saint Quentin Fallavier, France), unless otherwise stated in the text. Rabbit polyclonal antibodies to rat P44/42 mitogen-activated protein kinase (MAPK ERK1/2), human phospho-p44/42 MAPK (ERK1/2) (Thr202/Tyr204) were obtained from Cell Signaling Technology (Ozyme, Saint Quentin Yvelines, France). Mouse monoclonal antibodies to human vinculin (VCL clone hVIN-1) were purchased from Sigma-Aldrich. The HRP-conjugated anti-rabbit and anti-mouse IgG were purchased from Perkin Elmer (Courtaboeuf, France). Goat anti-rabbit antibodies conjugated with Alexa Fluor 488 were purchased from Life Technologies (Saint Aubin, France). The FFAR4 antibody was produced by rabbit immunization using a peptide designed from the second extracellular loop of the bovine sequence. Immunized rabbit serum underwent affinity purification, and the antibody concentration was measured by ELISA (Agro-Bio, la Ferté Saint-Aubin, France). Specificity of this FFAR4 antibody has been assessed (data not shown).

### Biological material

Bovine immature oocyte-cumulus complexes (OCC) were retrieved from bovine ovaries collected at a local slaughterhouse. Antral follicles from 3 to 6 mm in diameter were punctured using an 18G needle linked to a vacuum pump and to a 50 mL Falcon tube. OCC with compact cumulus layers were selected and washed in TCM 199 HEPES medium complemented with 0.04% BSA and 25 μg/mL gentamycin.

### Immunofluorescence

Immature oocytes with a partially reduced cumulus were fixed for 30 min in 4% paraformaldehyde diluted in PBS (phosphate buffer saline) pH 7.4. Following 15 min of incubation in 0.1% Triton/PBS, oocytes were incubated in PBS containing 5% goat serum and 2% bovine serum albumin (BSA). Samples were then incubated at 4 °C overnight either with rabbit FFAR4 antibody (9.5 ng/mL) or pre-immune serum (similar IgG concentration) of the same rabbit (negative control), both diluted in PBS/0.5% BSA. After washing four times for 30 min in PBS/0.1% BSA, the oocytes were incubated for 3 h in the dark with the secondary antibody, Alexa Fluor488 conjugated goat anti-rabbit IgG. Another four 30 min washes with PBS/0.1% BSA were performed, then OCC were incubated with Hoechst 33,258 (1 μg/mL) for 15 min and mounted on slides using Moviol® mounting medium. Green (Alexa 488) and blue (Hoechst) fluorescence was observed with a Zeiss confocal microscope (LSM700; Carl Zeiss Microscopy GmbH, Munich, Germany) using a 20× objective or an oil 63× objective and the appropriate filters. The images were collected with Zen 2012 software (black edition version 8.0, Carl Zeiss Microscopy GmbH).

### In vitro maturation (IVM)

IVM was performed with groups of 50–60 OCC in 500 μL of TCM199 supplemented with 10% (*v*/v) fetal calf serum (FCS), EGF (5 ng/mL), 17 β-estradiol (1 μg/mL), FSH (10 μg/mL), LH (12 μg/mL) (both Reprobiol, Lièges, Belgium) and gentamycin (5 μg/mL) in the presence or absence of DHA (1 μM) or TUG-891 (1 or 5 μM) (Tocris Bioscience, Bristol, United Kingdom) at 38.8 °C in a humidified atmosphere containing 5% CO_2_. DMSO (diluted 1:2000) was used as a control treatment because it was the solvent for DHA and TUG-891.

After 3, 4, 10 or 22 h of IVM, OCC were removed and oocytes and CC were kept at −80 °C until analysis. Oocytes were used for the analysis of the lipid composition and CC for gene expression studies.

### Transcriptome analysis

- *RNA extraction and labeling*

Four independent samples of CC collected after 4 h IVM in presence or absence of DHA 1 μM were used for transcriptomic analysis by microarray hybridization. Briefly, total RNA was extracted from CC using TriZol reagent (Invitrogen, Cergy Pontoise, France) and treated using RQ1 DNAse (Promega, Charbonnières, France) following the manufacturer’s instructions. After isopropanol precipitation, the RNA concentration was determined using a NanoDrop ND-1000 spectrophotometer (Nyxor Biotech, Paris, France) and RNA integrity was checked on a Bioanalyzer 2100 (Agilent Technologies, Santa Clara, CA, USA).

b) *Microarray hybridization and processing*

A customized 60 K bovine microarray (Agilent Technologies, Santa Clara, CA, USA, GEO profile GPL21724) covering more than 97% of Ensembl *Bos taurus* transcripts (genome assembly UMD3.1) was used. Cyanine-3 (Cy3) labeled cRNA were prepared with 200 ng of total RNA using the One-Color Low Input Quick Amp Labeling kit following the recommended protocol (Agilent Technologies, Santa Clara, CA, USA). Specific activities and cRNA yields were determined using the NanoDrop ND-1000. For each sample, hybridization was performed using 600 ng of Cy3-labeled cRNA (specific activity >6.0 pmol of Cy3/μg of cRNA) during 17 h at 65 °C, following the manufacturer’s protocol at the CRB GADIE facility (INRA Jouy-en-Josas, France, http://crb-gadie.inra.fr/). Slides were washed twice for 1 min at room temperature in GE Wash Buffer 1 and 1 min at 37 °C with the GE Wash Buffer 2 (Agilent Technologies) and air-dried. The slides were scanned immediately after washing using a G2565CA Scanner System with one color scan setting for 8 × 60 k array slides (Agilent Technologies). The resulting images were analyzed using Feature Extraction Software v10.7.3.1 (Agilent Technologies). The microarray data were submitted to the public repository Gene Expression Omnibus (GEO accession number GSE94338).

c) *Analysis of microarray expression data*

Raw data were normalized through intra-array median subtraction and log2 transformation using R statistical software [26].

Data were analyzed with a mixture model variance (VarMixt method [27]; raw probability values were adjusted using the Benjamini and Hochberg corrections [28]. As no gene was differentially expressed using this analysis and corrections, some genes (all correspondent probes of a given gene presented the same-sense variations with raw *p*-value <0.05 and fold change >1.5) presenting the highest fold-change and a significant raw p-value were selected and assessed by RT-PCR to confirm the absence of difference in gene expression.

d) *Real time RT-PCR analysis of gene expression in CC*

Total RNA was extracted as described above (part a). RNA preparations were DNAse digested by RQ1 DNase (Promega, Charbonnières, France) following the manufacturer’s instructions. Reverse transcription (RT) was performed on 200 ng of total RNA extracted from CC using the Maxima First Strand cDNA Synthesis kit (Thermo-Fisher Scientific, Courtaboeuf, France) according to the manufacturer’s instructions. Real-time PCR reactions were carried out on a CFX96 (Bio-Rad, Marnes-la-Coquette, France) in 20 μL containing primers at a final concentration of 150 nM each, 5 μL of the diluted RT reaction (2 ng cDNA) and qPCR Mastermix Plus for SYBR Green I (Bio-Rad, Marnes-la-Coquette, France) according to the manufacturer’s instructions. The efficiency of the primers and standard curve for each gene was deduced from serial dilutions of the correspondent cDNA fragment obtained as a template (Table 1). The relative gene expression levels were calculated in five to ten independent CC samples for each condition. The geometric mean of three housekeeping genes (*RPL19*, *RPS9* and *GAPDH*) was used to normalize gene expression. The relative amounts of gene transcripts (R) were calculated according to the equation: R = gene E^*-Ct gene*^/geometric mean (*RPS9* E^*-Ct RPS9*^ *; RPL19* E^*-Ct RPL19*^ *; GAPDH* E^*-Ct GAPDH*^ *)*, where E is the primer efficiency and Ct the cycle threshold.

**Table 1:** Oligonucleotide sequences

abbrev.	accession number	gene	Forward primer	Reverse primer	bp	E %
*AHCY*	NM_001034315	adenosylhomocysteinase	TGGACCCACCCAGACAAGTA	CAAAGGGCAGGTCTGGTTCT	202	95.8
*DHRS1*	NM_001046294	dehydrogenase/reductase (SDR family) member 1	GGGGGCTGCACTATCTCTTC	AATAATCCTGGACAGGGCGG	366	87.1
*EEF1G*	NM_001040487	eukaryotic translation elongation factor 1 gamma	CCAAGGATCCCTTTGCCCAT	GGAGAGCGTATCCTCGTTGG	86	93
*EIF2S1*	NM_175813	eukaryotic translation initiation factor 2, subunit 1 alpha	GTGGGACCTTGTTGTGGGAT	AGCTGACATAAGCGCCCATT	144	97.1
*SLC2A1 = GLUT1*	NM_174602	solute carrier family 2 (facilitated glucose transporter), member 1	CTGATCCTGGGTCGCTTCAT	ACGTACATGGGCACAAAACCA	68	91.4
*GAPDH*	NM_001034034	Glyceraldehyde 3 phosphate dehydrogenase	TTCAACGGCACAGTCAAGG	ACATACTCAGCACCAGCATCAC	119	98
*GPX1*	NM_174076	glutathione peroxidase 1	GCAACCAGTTTGGGCATCA	CTCGCACTTTTCGAAGAGCATA	116	87.3
*GPX4*	NM_174770	glutathione peroxidase 4	CGATACGCCGAGTGTGGTTTAC	ACAGCCGTTCTTGTCAATGAGG	261	89.8
*GSN*	NM_001113284	gelsolin	GGACCAGGTCTTTGTCTGGG	GATTAGCTGGGTCCGTCTCG	101	92.1
*FFAR4*	XM_865266	free fatty acid receptor 4	TTTCACTGCTGCTTCTTCGC	TTTCTGAGAGCCGGTACCCT	197	87.9
*FFAR1*	XM_002694932	free fatty acid receptor 1	GCCATCGTTCTCTGTCACCT	CCGGAGAGCCATTGATTGGT	112	87.5
*Met_SRP*	ENSBTAT00000065876	Metazoan signal recognition particle RNA	CGCCTGTAGTCCCAGCTACT	ATCAGCACGGGAGTTTTGAC	211	90.1
*MTMR3*	NM_001081605	myotubularin related protein 3	CTGCGAAAAGCTGCTAACCG	CCTGAGACACAACTTGCCCT	119	90.8
*RPL19*	BC102223	Ribosomal protein L19	AATCGCCAATGCCAACTC	CCCTTTCGCTTACCTATACC	156	97.3
*RPS9*	BC148016	Ribosomic protein S9	GGAGACCCTTCGAGAAGTCC	GGGCATTACCTTCGAACAGA	180	94.2
*SIRT2*	NM_001113531	sirtuin 2	CTGGCCAGACTGACCCTTTC	CTTCCATCCAAGGAGGTCGG	146	91.3
*SNORA16*	ENSBTAT00000059978	Small nucleolar RNA SNORA16B/SNORA16A family	CGTGGCCCTTATCGAAGCTG	GCGACCGTCAAGGAAAACTG	87	90.6
*SNORA17*	ENSBTAT00000059474	Small nucleolar RNA SNORA17	AGAGGAAAGAGGCTCGGTCT	AGTGTCAGACTATCAATCATCCAGA	71	82.8
*SNOU6_53*	ENSBTAT00000059571	Small nucleolar RNA U6–53/MBII-28	GTTGCCATGCTAATACTGAGCC	TGTTAAACTCACTGGCACCCA	70	94.3
*U1*	ENSBTAT00000051513	U1 spliceosomal RNA	CACGAAGGTGGTTTTCCCTA	GCCCCCACTACCACAAGTTA	114	110
*VCP*	NM_001034294	valosin containing protein	TGCTTGCAAGTTGGCCATTC	GCAAAGCGCATAGCTTCCTC	150	83.5

### Lipid profiling of cumulus cells and single oocytes by MALDI-TOF mass spectrometry

After 24 h IVM with or without DHA at 1 μM or TUG-891 at 5 μM, CC were separated from the oocytes by aspiration-rejection movements using a micropipette, centrifuged (3500 x g for 5 min) and washed in PBS pH 7.4 and then in Tris-sucrose (TS) buffer (20 mM Tris-HCl, 260 mM sucrose, pH 6.8). After final centrifugation, TS was discarded and CC samples were immediately frozen at −80 °C. Single denuded oocytes were rinsed in two successive drops of TS buffer and then frozen individually in 5 μL of the same buffer. CC and oocytes samples were kept at −80 °C before performing the lipid profiling using MALDI-TOF MS. Experiments were performed twice using 12 OCC per condition (control IVM, IVM with 1 μM DHA and IVM with 5 μM TUG-891); four independent pools of CC (from three OCCs each) and 12 individual oocytes were analyzed in each experiment. All cumulus or oocytes samples from the same experiment were analyzed by MS the same day.

Fresh matrix solution containing 20 mg/mL of 2,5-dihydroxyacetophenone (DHAP) in 90% methanol, 10% H_2_O in presence of 2% trifluoroacetic acid (TFA) was prepared just before analysis. Oocytes were loaded individually onto an MTP Ground Steel 384 MALDI sample plate (Bruker Daltonics, Germany), then the excess TS buffer was removed and each oocyte was overlaid with 1.5 μL of DHAP matrix and air-dried.

Four microliters of DHAP matrix was added in each CC sample tube and vortexed for 30 s. Sample were then sonicated using an ultrasonic bath sonicator (FisherBrand 15,052, Fisher Scientific) to solubilize lipids, for 3 min in iced water. One microliter of CC suspension was spotted in duplicate onto the MALDI sample plate and overlaid with 1.5 μL of DHAP matrix and air-dried.

The lipid spectral profiles from each oocyte or CC sample were acquired using an UltrafleXtreme MALDI-TOF/TOF instrument (Bruker Daltonics, Bremen, Germany) controlled by FlexControl 3.0 software (Bruker Daltonics, Germany), equipped with a Smartbeam laser at a 2 kHz laser repetition rate. The spectra were obtained in reflectron mode using the negative and positive ion modes, in the 100 to 1000 m/z range, and collected as a sum of 1000 laser shots in three shot steps (total of 3000 shots per spectra). The parameters used for spectra acquisition were: ion source 1, +25.23/−20.18 kV; ion source 2, +22.45/−18.04 kV; lens, +8.01/−6.70 kV; pulsed ion extraction at 120 ns; and laser focus parameter set at medium, respectively for the positive and negative ion modes. External calibration was followed using a “home-made” calibrant solution (1 μL of calibrant solution plus 1 μL of matrix) containing caffeine, MRFA peptide, bradykinin 2–9, bradykinin, angiotensin I and Glu1-fibrinopeptide B, all at 2 pmol/μL. A quadratic algorithm was applied to obtain a deviation <11 ppm.

For each CC sample, two spots were analyzed and spectra were acquired in triplicate (a total of six spectra replicates for each CC sample). One spectrum (3000 shots) per oocyte was acquired.

FlexAnalysis version 3.4 (Bruker Daltonics, Bremen, Germany) was employed to extract spectral data and to generate txt files. Data were integrated in Progenesis MALDI™ version 1.2 (Nonlinear Dynamics, Newcastle upon Tyne, UK) and treated for baseline subtraction (Top Hat filter 60), denoising (Noise filter 4) and spectra alignment. Automatic peak detection was applied to the reference spectrum (a weighted average of all experimental spectra) in the range of 200 to 1000 m/z with a threshold fixed at 150 counts corresponding to a signal to noise ratio > 1.5. Normalization on peak height was performed using the Total Ion Count (TIC) in order to display and compare all spectra on the same scale.

The reproducibility of spectral analysis linked directly to the spectrometer process was evaluated by the coefficient of variation (CV) of six technical replicates for all the peaks of all CC samples. Mean CV values were 37.1 ± 1.0% in positive mode and 28.1 ± 0.5% in negative mode, according to all peak values.

### Lipid identification by high-resolution mass spectrometry

After Folch lipid extraction of bovine follicular cells, the lipid extract was reconstituted in methanol:water (*v*/v) in the presence of formic acid and directly infused into a high-resolution mass spectrometer (LTQ Orbitrap Velos, Thermo Fisher Scientific, Bremen, Germany). Standard mass spectrometric conditions for all experiments were: spray voltage 1.2 kV, no sheath and auxiliary gas flow; heated capillary temperature, 250 °C; predictive automatic gain control (AGC) enabled, and an S-lens RF level of 60%. Data were acquired using Xcalibur software (version 2.1; Thermo Fisher Scientific, San Jose, CA). The LTQ Orbitrap Velos instrument was operated in positive mode in data-dependent mode to automatically switch between full scan MS spectra and MS/MS. Resolution in the Orbitrap was set to *R* = 100,000. In the scan range of m/z 400–1800, the 10 most intense mono-charged ions were sequentially isolated and fragmented by HCD, with normalized collision energy of 38% and wideband-activation enabled. The ion selection threshold was 500 counts for MS/MS. Target ion quantity for FT full MS was 1 × 10^6^ and for MS2 was 1 × 10^4^. An activation q = 0.25 and activation time of 10 ms were used. Dynamic exclusion was activated during 30 s with a repeat count of 1. Raw data files were converted to MGF with Proteome Discoverer software (version 1.2; Thermo Fischer Scientific, San Jose, USA).

Identification of lipids was performed using Lipid Blast software (Metabolomics Fiehn Lab, 240,313 sprectra in 13 libraries). The search tolerance used for precursor ion masses was 0.4 m/z and for theoretical fragment ions masses was 0.8 m/z. Reported compound identifications were manually validated using fragment ion matches based on the putative chemical structures.

### In vitro fertilization (IVF) and analysis of in vitro embryo development

IVF was performed as previously described [15]. The cleavage rate was assessed at day 2 after fertilization and embryo development was checked at day 7 post-IVF. Experiments were repeated five times with 50–60 OCC per each IVM condition per experiment.

At day 2 of in vitro development (IVD), embryos were observed using a Zeiss inverted microscope (Zeiss, Germany) in order to count cell numbers in the cleaved embryos, and then in vitro culture was continued. Embryos were analyzed 7 days after the beginning of IVF (168 h), and the number of total blastocysts and expanded blastocysts was reported. All embryos were fixed in 4% paraformaldehyde and stained with Hoechst 33,342 (1 μg/mL). The number of cells in each embryo was counted using an Axioplan Zeiss fluorescent microscope. Degraded embryos without visible chromatin staining were not taken into account. The blastocyst rate was defined as the total number of blastocysts reported in the number of cleaved embryos. The expanded blastocyst rate was defined as the total number of expanded blastocysts reported in the number of cleaved embryos.

### Western blot analysis

Proteins were extracted from the oocytes (groups of 30) or CC (groups of 10 OCC) as previously described [29]. Denatured proteins were resolved by 4–12% SDS-PAGE (Life technologies, Saint-Aubin, France), transferred onto a nylon membrane, blocked with 5% of milk powder in Tris-buffered saline with 0.1% Tween 20 for 1 h at room temperature and probed with different antibodies overnight at 4 °C. Antibody dilutions were 1:1000 for phospho-MAPK3/1 and total MAPK3/1. Immunoreactivity was detected using anti-rabbit HRP-conjugated secondary antibodies (diluted 1:10,000), revealed using enhanced chemiluminescence ECL (West Dura; Thermo Scientific, Courtaboeuf, France) and quantified using a GeneGnome charge-coupled device camera (Syngene, Cambridge, United Kingdom) with Genesys 1.5.4 software (Syngene). Analysis of signal intensity was performed with GeneTools 4.01 software (Syngene). Four independent samples of oocytes or OCC per IVM condition were analyzed.

### Statistical analysis

For comparison of lipid profiles between the groups of control and treated oocytes (*n* = 15 per group), Student’s t-test was applied to log values of normalized peak heights; differences were considered significant at *p*-values <0.01. The non-parametric Mann-Whitney test was used to compare relative lipid abundance in CC samples (*n* = 4 per group). Multivariate principal component analysis (PCA) and hierarchical clustering were performed for the assessment of lipid content variations under different conditions using XLSTAT 3.01 (Addinsoft, Paris, France).

Statistical analyses taking into account both treatment effect and replica effect were performed for all parameters of embryo development. The distribution of cleavage rates and blastocyst rates were compared by logistic regression analysis using a generalized linear model (R package Rcmdr, [30] and R version 3.2.1, [26]). Least square means (lsmeans) estimated by the models (R package lsmeans, [31]) were subsequently compared to the control conditions. Results are presented in tables and figures as lsmeans ± SEM, unless otherwise stated.

The number of blastocyst cells was compared between the groups using either parametric ANOVA, when the distribution and variance enabled performing a parametric study (Shapiro test, Levene test, Rcmdr package) with a Tukey post hoc comparison (multcomp package), or non-parametric ANOVA (permutational ANOVA), when the distribution was not normal and the variance was not homogenous (R package lmPerm, [32] with the Tukey post-hoc test (R package nparcomp, [33] and R version 3.3.1, [26]). Real-time PCR and Western blotting data were compared between the groups using non-parametric ANOVA. A difference with *p* ≤ 0.05 was considered significant and with 0.05 < *p* ≤ 0.10 was considered a tendency.

## Results

### Effect of 1 μM DHA on gene expression in cumulus cells during IVM

**Fig. 1:**
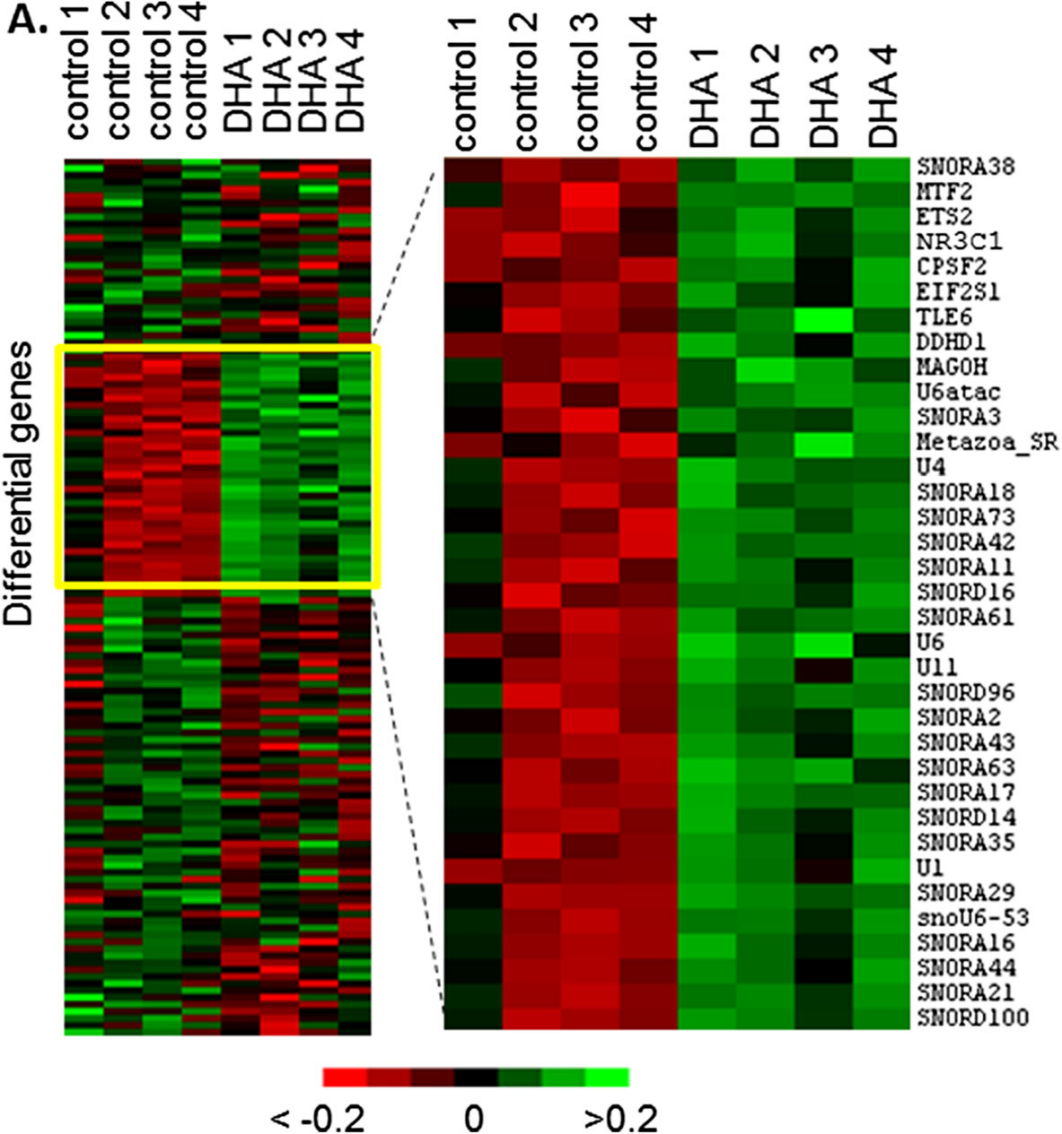
Analysis of gene expression in cumulus cells of control and DHA 1 μM-treated oocytes during IVM. **a**. Heatmap representation of differentially expressed genes (raw *p*-value <0.05) between control and DHA 1 μM-treated cumulus cells, as detected by analysis of microarray hybridizations. *Zoom*: cluster of up-regulated genes in the presence of 1 μM DHA

In order to decipher the possible mechanism of the beneficial effect of 1 μM DHA on oocyte quality, we compared the transcriptomic profiles of control and DHA-treated cumulus cells after 4 h IVM by microarray. Comparative analysis using mixture model variance and a Benjamini-Hochberg correction showed no differential genes with an adjusted *p*-value <0.05. In order to confirm the absence of difference in gene expression, genes exhibiting raw *p*-values <0.05 for all the oligonucleotide probes of the same gene were considered (all correspondent probes presenting the same-sense variations) and provided a list of 127 genes: 35 genes were up-regulated and 92 down-regulated by 1 μM DHA (Additional file 1: Table S1). Using hierarchical clustering, the genes exhibiting the same expression pattern were grouped and data were presented here as a heatmap (Fig. 1). Only four genes were decreased by more than two-fold (*GSN, LLGL1, MTA1* and *GRO1*) and 10 genes were increased by more than two-fold (*SNORA16, SNORA17, SNORA21, SNORA29, SNORA35, SNORA44, SNORD14, SNORD100, U1*, and *SNOU6–53*) in DHA-treated samples compared to the control.

Among the 35 up-regulated genes in DHA treated CC, 20 genes corresponded to small nucleolar RNA and five genes corresponded to spliceosomal RNA.

**Fig. 2:**
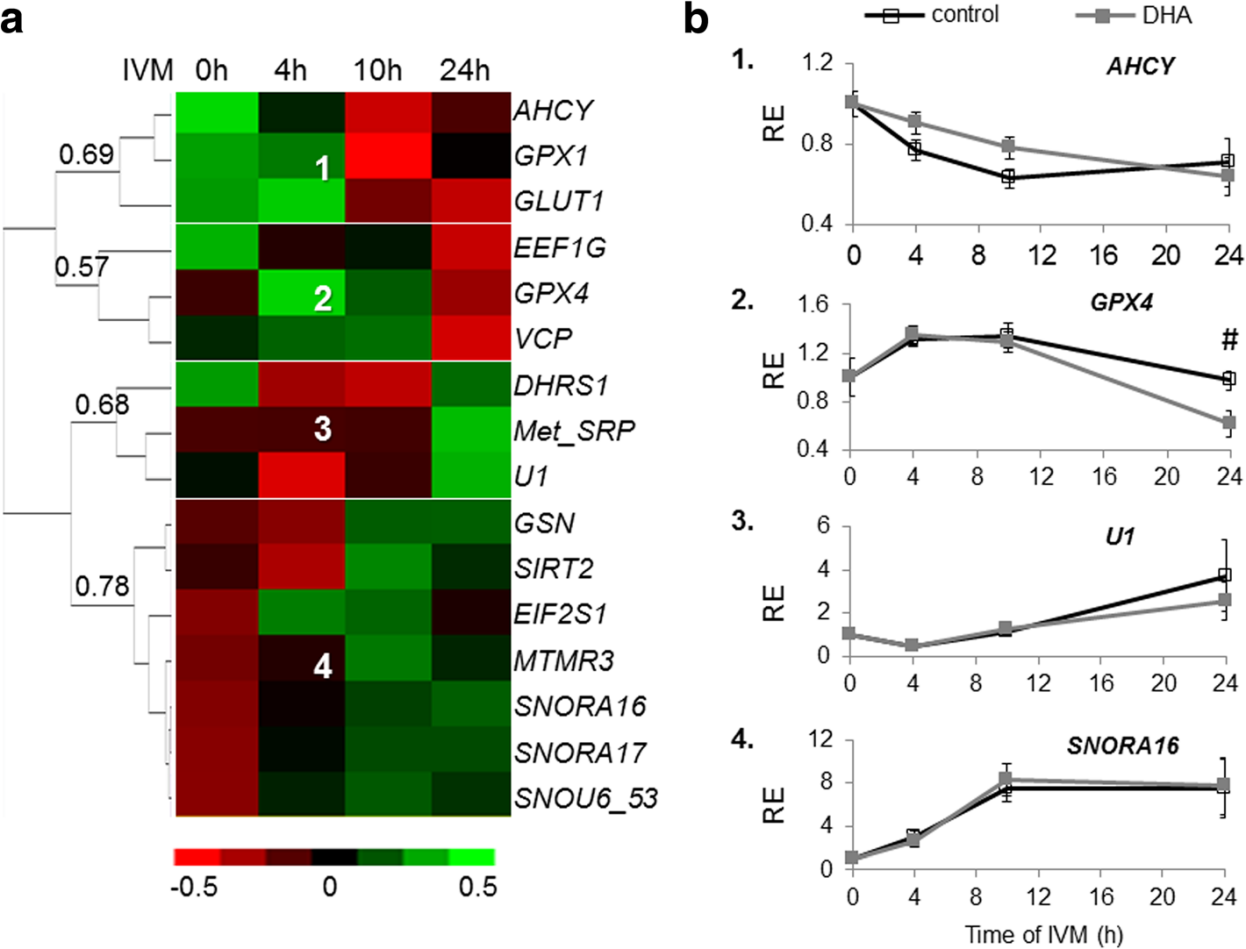
Real-time PCR quantification of the expression of several differential genes. **a.** Heatmap clustering representation of expression kinetics of several differential genes during IVM. **b**. Comparative expression of representative genes from each cluster, in control and 1 μM DHA-treated samples during IVM. Eight independent CC samples were analyzed per group. # indicates tendency toward a difference from the control for the same time point (0.05 < *p* < 0.1)

We used real-time PCR to analyze expression kinetics of 13 genes differentially expressed according to microarray and three candidate genes (previously reported to be regulated by DHA) involved in glucose metabolism (*GLUT1*) or in oxidative stress (*GPX1* and *GPX4*), in the absence or presence of 1 μM DHA after 4 h, 10 h and 24 h IVM (Additional file 2: Table S2). A hierarchical clustering defined four clusters, according to four expression patterns during IVM (Fig. 2a). Clusters 1 and 2 included four genes showing a significant decreased expression throughout maturation (*AHCY, GPX1, GLUT1* and *EEF1G*) (Additional file 2: Table S2). On the contrary, in clusters 3 and 4, seven genes showed a significant increased expression throughout maturation (*Met-SRP, GSN, SIRT2, MTMR3, SNORA16, SNORA17* and *SNOU6–53*). No significant differences were found at 4 h and 10 h IVM between DHA-treated and control groups, in all the genes analyzed (*p* < 0.05). Nevertheless, *GPX4* showed a tendency toward decreased expression in 1 μM DHA treated cells compared to control (*p* = 0.055) at 24 h IVM (Fig. 2b). Therefore, 1 μM DHA supplementation during IVM did not significantly change the transcript level of individual genes and thus confirmed the microarray analysis reporting no significant difference in gene expression.

### Effect of 1 μM DHA on the lipid composition of oocytes and CC after 24 h IVM

**Fig. 3:**
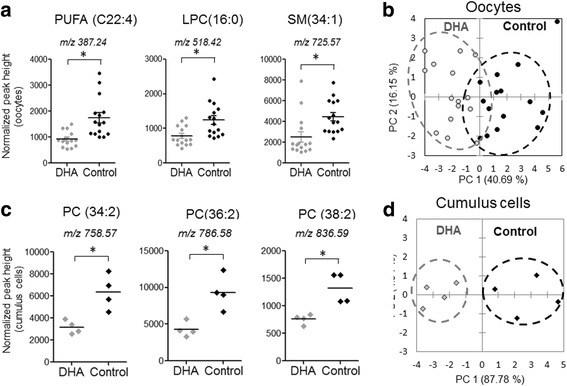
Analysis of lipid composition using MALDI-TOF mass spectrometry profiling in bovine oocytes and cumulus cells after 24 h IVM with or without DHA 1 μM treatment. Individual oocytes (15 per group) and triplicates of pools of CC (4 per group) were analyzed. Relative abundance of lipid species in DHA-treated and control oocytes (**a**) and cumulus cells (**c**). Principle component analysis and scatter-plots of differential lipids in control vs. DHA-treated oocytes (**b**) and in cumulus cells (**d**)

Comparative analysis of the lipid profiles was performed using MALDI-TOF mass spectrometry on individual intact oocytes and corresponding CC after 24 h IVM in the presence or absence of DHA 1 μM. Using positive acquisition mode (200–1000 m/z), 324 and 406 peaks were detected in CC and in the oocytes, respectively (Additional file 3: Table S3). Using negative acquisition mode (200–1000 m/z), 374 (in CC) and 294 peaks (in oocytes) were detected (Additional file 3: Table S3). Using differential analysis of lipid spectra (both positive and negative modes) between control and DHA-treated oocytes (*n* = 15 per condition), only three peaks out of 700 (m/z 387.24, 518.42 and 725.57) showed significantly lower abundance in DHA-treated oocytes (*p* < 0.01, FC > 1.5) and m/z 965.47 increased in DHA-treated oocytes: (Fig. 3a, Additional file 4: Table S4). Peaks with m/z 518.42 and m/z 725.57 were formerly identified as LPC 16:0 + Na^+^ and SM 34:1 + Na^+^ (Additional file 5: Table S5) and m/z 387.24 was annotated as potentially being an unsaturated fatty acyl. Principle component analysis discriminated the control and DHA groups; however, they partially overlapped (Fig. 3b).

Lipid profiles of CC differed in seven peaks between the two groups, from 698 m/z detected (p < 0.01, fold-change >1.5, Additional file 4: Table S4). The relative abundance of these seven lipids was significantly lower in DHA-treated cumulus cells compared to control (Fig. 3 c). The four most abundant m/z were previously identified as dipolyunsaturated phosphatidylcholine (PC): m/z 758.57 PC 34:2 + H^+^, m/z 784.56 as PC 34:0 + Na^+^, m/z 786.58 as PC 36:2 + H^+^ and m/z 836.59 as PC 38:2 + Na^+^. Control and DHA groups were clearly discriminated by PCA (Fig. 3d).

Therefore, 1 μM DHA supplementation during 24 h IVM led to moderate changes in the lipid composition of oocytes and CC.

### FFAR4 in oocytes and cumulus cells

In order to investigate whether DHA could act through membrane receptors, we first analyzed the expression of two free fatty acid receptors, FFAR1 and FFAR4, in bovine oocytes and CC. *FFAR4* transcripts were repeatedly detected in CC by real time RT-PCR and its expression level was higher than that of *FFAR1*: normalized expression values measured on 50 ng cDNA per reaction were 1.2 10^−4^ ± 3.8 10^−5^ (*n* = 2) and 1.5 10^−5^ ± 8.6 10^−6^ (n = 2), respectively. As FFAR4 seemed to be expressed at a higher level than FFAR1, we chose this receptor for further investigation in this study.

**Fig. 4:**
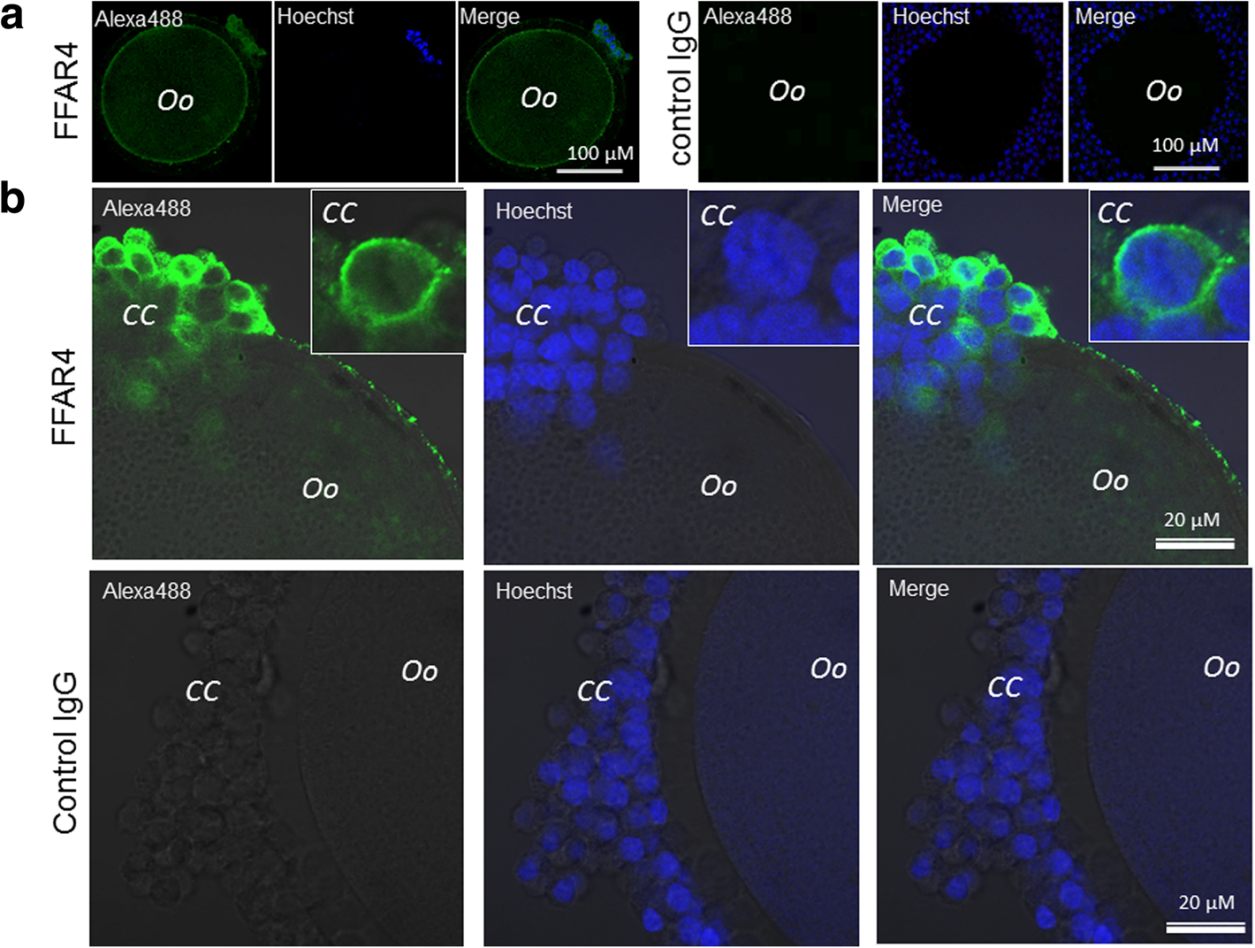
Intracellular localization of FFAR4 protein in the bovine oocyte-cumulus complex by immunofluorescence. Alexa488 green fluorescence indicates FFAR4 localization to the vicinity of cell membrane in the oocyte (**a**) and in cumulus cells (**b**). Rabbit IgG from before immunization was used as the negative control (IgG). Hoechst blue staining indicates CC nuclei

FFAR4 protein was detected by immunofluorescence in both CC and oocyte using a customized specific antibody against bovine FFAR4 protein (Fig. 4). The green fluorescence corresponding to FFAR4 protein was concentrated preferentially at the periphery of both oocytes (Fig. 4a) and CC (Fig. 4b), which likely corresponded to the cell membrane. Different CC showed different intensities of FFAR4 immunostaining, and only cytoplasm but not nuclear staining was observed in CC. The labelling intensity of FFAR4 suggested a higher expression of the receptor FFAR4 in the cumulus cells than in the oocyte (Fig. 4b).

Thus, the membrane receptor FFAR4 was present and localized to the vicinity of cellular membrane in both oocytes and surrounding CC.

### Comparative analysis of the FFAR4 agonist, TUG-891, and DHA supplementation during IVM on in vitro embryo development

**Fig. 5:**
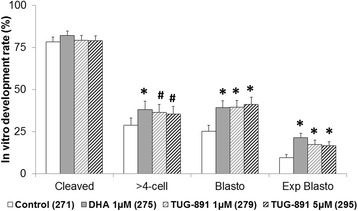
In vitro developmental rates of bovine oocytes treated with either DHA 1 μM, or TUG-891 1 μM, or TUG-891 5 μM or control during 24 h IVM. The cleavage rate was checked at day 2 and the blastocyst rate at day 7 after in vitro fertilization and are presented as mean ± SEM of 5 independent experiments. * indicates a significant difference from the control (*p* < 0.05), # indicates a tendency towards a difference from the control (0.05 < *p* < 0.1)

DHA 1 μM and TUG-891 1 μM or 5 μM, supplemented during IVM for 24 h, showed no effect on the embryo cleavage rate (82.1 ± 2.7, 79.3 ± 2.9 and 79.1 ± 2.8, respectively) compared to the control (78.3 ± 3.0%) at day 2 after IVF (Fig. 5). No effect of the treatments was observed on the 2 to 4-cell embryo rate. However, DHA 1 μM led to a significant 1.32-fold increase (*p* = 0.023) in the rate of >4-cell embryos at day 2 (38.2 ± 4.8% versus 28.9 ± 4.3% for the control); TUG-891 1 and 5 μM followed the same tendency (36.5 ± 4.7%, *p* = 0.061 and 35.4 ± 4.6%, *p* = 0.100, respectively). At day 7 after IVF, blastocyst rates were significantly increased by DHA 1 μM (1.56-fold increase, *p* = 0.002), TUG-891 1 μM (1.57-fold increase, p = 0.002) and 5 μM (1.64-fold increase, *p* = 0.001) compared to the control. Expanded blastocyst rates were also significantly increased compared to the control with DHA 1 μM (2.24-fold increase, p = 0.001), TUG-891 1 μM (1.82-fold-increase, *p* = 0.019) and TUG-891 5 μM (1.76-fold increase, *p* = 0.026).

The number of cells per blastocyst at Day 7 post-IVF were similar between these conditions (control 96.5 ± 5.0 cells, DHA 1 μM 110.4 ± 4.8 cells, TUG-891 1 μM 106.9 ± 4.9 cells and TUG-891 5 μM 100.9 ± 4.4).

Therefore, DHA 1 μM and TUG-891 1 or 5 μM similarly increased the embryo developmental rate.

### MAPK3/1 phosphorylation in DHA 1 μM treated cumulus cells and oocytes

**Fig. 6:**
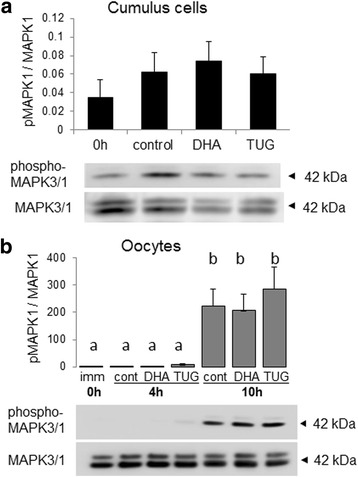
Western blot analysis of phospho-MAPK3/1 in DHA 1 μM treated or control CC or oocytes. (**a**) MAPK3/1 phosphorylation in CC after 3 h IVM; (**b**) MAPK3/1 phosphorylation (**b**) in control and DHA-treated oocytes at 4 h and 10 h IVM. 10 COCs (**a**) or 30 oocytes per line (**b**) were loaded. Four independent samples per condition were analyzed. Different letters indicate significant differences at *p* < 0.05

Phosphorylation of MAPK3/1 was investigated in DHA and TUG-891 1 μM treated or control CC (after 3 h IVM) or oocytes (after 4 h and 10 h IVM) (Fig. 6). No difference in MAPK3/1 phosphorylation was observed in CC after 3 h IVM between conditions. Concerning MAPK3/1 phosphorylation in oocytes, a huge significant increase (more than 100-fold) is observed between 4 h and 10 h IVM. Nevertheless, no difference was observed between conditions, at any time.

## Discussion

In the present study, we performed experiments to decipher the possible mechanisms of action of low dose DHA during IVM culture, which could improve oocyte competence to better support in vitro embryo development in cattle [15]. DHA has already been reported to be able to affect cellular functions through several mechanisms: transcription factor regulation, eicosanoid production, membrane lipid composition modification or through free fatty acid receptor binding [17]. We therefore compared DHA-treated oocytes and surrounding CC by analyzing lipid composition and MAPK3/1 phosphorylation during IVM in both compartment and gene expression in CC. We also tested the hypothesis that the membrane receptor FFAR4 may be involved in the mechanism of action of DHA and therefore we performed functional studies using a relevant pharmacological agonist, TUG-891.

### DHA supplementation during IVM moderately affected the cumulus cell transcriptome

Overall, according to the microarray data, cumulus gene expression after 4 h IVM was not affected by addition of low concentration of DHA (1 μM). Absence of effect of DHA at transcript level is possibly due to that 4 h of IVM was too short to observe transcriptional modifications in our model. Nevertheless, even when looking at a longer IVM period, the expression of canonical genes already described in adipocytes or other cell types as being affected by n-3 PUFA did not change in CC under our conditions [15]. Transcriptional effect of DHA-derived oxylipins was previously reported with a higher DHA concentration, i.e. 10 μM in human macrophages [34] and rat brain endothelial cells [35], 80 μM in lung epithelial cells [36] or from 25 to 200 μM in adipocytes [37]. The transcriptional effects of DHA are not always detected even on canonical genes such as *PPARG* [38], as in our present study. Moreover, effects on signaling pathways or on PPARG translocation were also observed at higher DHA concentrations, i.e. 20 μM in human gastric cancer cells [39]. The DHA concentration of 1 μM used in this study was previously compared to higher concentrations (10 and 100 μM) and shown to exert beneficial effects on oocyte competence and embryo development [15].

In our study, the only transcriptional difference we observed, was a tendency of a decrease in *GPX4* expression in DHA-treated cumulus cells, suggesting a reduced oxidative stress environment after 24 h of treatment. Such an effect on *GPX4* expression has also been reported in mouse hippocampal cells after receiving a high-DHA diet [40]. Despite the absence of effects of DHA on lipid metabolism genes previously reported [15], it is possible that DHA might affect energy production. Indeed, n-3 PUFA have previously been reported to increase lipolysis in adipose tissue, especially at the protein level [41]. Energy production should therefore be investigated. Nevertheless, despite the absence of DHA effect on their expression, the expression of some of the analyzed genes increased through IVM (*Met-SRP, GSN, SIRT2, MTMR3, SNORA16, SNORA17* and *SNOU6–53*), suggesting their potential involvement in CC during oocyte maturation.

### DHA supplementation during IVM changed some lipids in oocytes and CC

By comparing mature oocytes and their CC with or without DHA treatment during IVM, some modifications in the lipid composition were observed. In fact, we have previously shown that CC lipid fingerprints changed through IVM and were also affected by the inhibition of FA oxidation [42].

In this study, the reduction in lysophosphatidylcholine (LPC) observed in DHA treated oocytes may be relevant to a potential increase in lysophosphatidic acid (LPA), as LPC is a precursor of LPA. It has been reported that DHA could inhibit LPA-induced proliferation in breast or prostate cancer cells [43, 44]. It has already been described that LPA can improve bovine oocyte maturation [45]. A potential increase in LPA could therefore explain some of the beneficial effects of DHA on oocyte quality. As most of differential lipid species were decreased in DHA treated cells, we explored the lipolysis mechanism in these cells by exploring HSL activity and FABP3 expression. However, no differences in either HSL phosphorylation or in FABP3 protein abundance were observed between control and DHA-treated oocytes.

Globally, the changes observed in the lipid composition of CC and oocytes were limited, which could be explained by the very similar lipid composition (mainly brought by the calf serum) of the medium and exhibiting only a 1 μM DHA difference.

Overall, despite functional differences between DHA-treated and control oocytes, there were no huge changes in cumulus gene expression and the oocyte/CC lipid composition. These results suggest that these mechanisms are not critical to mediating the effects of DHA, especially given the timing of our experiment, which may support our hypothesis of a direct effect on signaling pathways, consecutively through FFAR4 binding.

### DHA effect on embryo development was partly reproduced by FFAR4 stimulation

For the first time, we show here that FFAR4 is expressed in both oocytes and CC. We demonstrated that FFAR4 was localized to the cellular membrane in both oocyte and CC, as expected for a G-protein coupled receptor. Moreover, FFAR4 stimulation by TUG-891 led to a similar increase in the blastocyst rate compared to DHA, suggesting that FFAR4 stimulation was able to partly reproduce the DHA effect on oocyte developmental potential in vitro, i.e. by increasing the in vitro embryo developmental rate. Indeed, TUG-891 is a specific and potent agonist of FFAR4 [25, 46]. Thus, the effects observed after TUG-891 treatment should be related to FFAR4 activation. It has already been described that DHA can exert effects through FFAR4 stimulation in adipocytes [47], in macrophages [48] or in cardia cells [49]. Moreover, several well-demonstrated DHA effects, such as the ability to inhibit the responsiveness of macrophages to endotoxin through the inhibition of IκB kinase phosphorylation, IκB phosphorylation and degradation, and TNF, IL-6 and MCP-1 production, were abolished in FFAR4 knockdown cells [19, 50].

Of note, blastocyst production was performed in suboptimal conditions. Indeed, selection of OCC only excluded denuded OCCs and those with either general expansion of the cumulus cells or discolored cytoplasm. This suboptimal selection of OCC explained the relatively low blastocyst rate of the control. The present study highlighted the beneficial effect of DHA and TUG-891 on the blastocyst rate when dealing with low quality oocyte. It is thus possible that, when dealing with high quality oocyte, the effect of DHA or TUG-891 would have been moderate or even absent.

Several days of TUG-891 treatment in 3T3L1 cultures has also been reported to modify PPARγ and α transcription [47]. Under our conditions, we analyzed gene expression of several genes in CC after 4 h of DHA (or up to 24 h [15]), so this treatment duration might be too brief to enable transcriptional modifications following FFAR4 stimulation.

Stimulation of FFAR4 leads to cell specific effects. Indeed, in human intestinal epithelial Caco-2 cells, FFAR4 was internalized, bound to β-arrestin-2, and attenuated NF-κB activation in response to 30-min exposure to the agonists GW9508, TUG-891, or to docosahexaenoic acid. However, treatment of mouse STC-1 intestinal epithelial cells with these agonists did not induce receptor internalization and had no effects on NF-κB activation [48].

In order to gain insight into the DHA mechanism in oocyte and CC, signaling pathways known to be affected by DHA should be investigated. In particular, the NFκB, JNK, PI3K, MAP kinase pathways may be involved. It is known that TUG-891100 μM leads to an increase in MAPK1/3 phosphorylation in 3 T3-L1 adipocytes [47]. Under our conditions, modification of MAPK1/3 phosphorylation was neither detected in oocytes nor in CC with low DHA or TUG concentrations at several time points of IVM, even though phospho-MAPK3/1 have increased in oocytes during IVM, similarly to previous studies [51]. This might be due to the lower TUG-891 concentration we used, thus leading to weaker FFAR4 stimulation. Therefore, further experiments are required to demonstrate that DHA is able to bind FFAR4 in bovine cells and to activate its signaling pathways (e.g. intracellular calcium assay). However, low FFAR4 expression level in the oocyte-cumulus complex makes it difficult to study changes in signaling due to DHA treatment or FFAR4 stimulation. For future studies, it is thus crucial to inactivate the receptor. However, in our model, consisting of oocyte and highly specialized cumulus cells, it would be rather difficult to inactivate this receptor in vitro. Signaling pathway studies, as in the literature, should be performed in models that could enable overexpression (plasmid transfection) and receptor inactivation in similar cell types like granulosa cells.

## Conclusion

DHA added at low physiological concentration (1 μM) during IVM is able to improve oocyte quality in vitro. FFAR4 activation is able to reproduce DHA effects on oocyte quality. DHA therefore might activate this receptor in CC and trigger signaling pathways leading to remote changes, thereby improving oocyte quality. Nevertheless, further experiments are needed to demonstrate FFAR4 activation by DHA. Intracellular lipid composition and transcriptional modulations might not be critical under our conditions to mediate the early effects of DHA. Future studies should focus on a more extensive characterization of the numerous signaling pathways to decipher their precise role and importance regarding the mechanism of action of DHA, and should analyze the effects of higher concentrations and/or longer DHA treatment periods.

## Additional files

Additional file 1: Table S1. Differencial genes on microarray analysis (raw *p*-value). (PDF 473 kb)

Additional file 2: Table S2. Cumulus gene expression during maturation in absence or presence of DHA 1 μM. Relative expression is expressed relatively to the geometric mean of 3 housekeeping genes (*RPL19*, *RPS9* and *GAPDH*) and presented as mean ± sem. (PDF 1677 kb)

Additional file 3: Table S3. Lipids detected by MALDI MS in bovine cumulus cells (CC) and oocytes (OO), in positive (+) et negative (−) modes. (PDF 609 kb)

Additional file 4: Table S4. Differential analysis of lipids in control and DHA 1 μM –treated oocytes and cumulus cells. (PDF 1215 kb)

Additional file 5: Table S5. Possible annotation of differential m/z peaks. (PDF 224 kb)

## Abbreviations

ALA: Alpha-linolenic acid
BSA: Bovine serum albumin
CC: Cumulus cells
DHA: Docosahexaenoic acid
DHAP: 2,5-dihydroxyacetophenone
DMSO: Dimethyl sulfoxide
DNA: Deoxyribonucleic acid
EGF: Epidermal growth factor
EPA: Eicosapentaenoic acid
FA: Fatty acids
FCS: Fetal calf serum
FFAR1: Free fatty acid membrane receptor 1
FFAR4: Free fatty acid membrane receptor 4
FSH: Follicle-stimulating hormone
GC: Granulosa cells
GLUT1: Glucose Transporter Type 1
GPX4: Glutathione peroxidase 4
HRP: Horseradish peroxidase
IgG: Immunoglobulin G
IL-1β: interleukine 1 beta
IL-6: Interleukine 6
IL-8: Interleukine 8
IVF: in vitro fertilization
IVM: in vitro maturation
IκB: I kappa B
LDL: Low density lipoprotein
LH: Luteinizing hormone
LPA: Lysophosphatidic acid
LPC: Lysophosphatidylcholine
MAPK1/3: Mitogen-Activated Protein Kinase 1/3
MCP-1: Monocyte Chemoattractant Protein-1
NFDMP: Non-fat dry milk powder
NFkB: Nuclear factor kappa B
OCC: Oocyte-cumulus complex
PBS: Phosphate buffered saline
PCA: Principal component analysis
PCR: Polymerase chain reaction
PPARs: Peroxisome proliferator-activated receptors
PUFA: Polyunsaturated fatty acid
RNA: Ribonucleic acid
RPL19: Ribosomal protein L19
RPS9: Ribosomal protein S9
SEM: Standard error of the mean
TBS: Tris-buffer saline
TFA: Trifluoroacetic acid
TGFβ1: Transforming growth factor beta1
TNF: Tumor necrosis factor
