# Supplementary material for: Docosahexaenoic acid mechanisms of action on the bovine oocyte-cumulus complex
Source: J Ovarian Res. 2017 Nov 9;10:74. doi: 10.1186/s13048-017-0370-z (PMC5679375; doi:10.1186/s13048-017-0370-z)
Supplement: Supplementary file 1 — Differencial genes on microarray analysis (raw p-value). (PDF 473 kb) [file 13048_2017_370_MOESM1_ESM.pdf]

Additional file 1: Table S1 : differential genes on microarray analysis (raw p-value)

All differential p<0.05

Ensemble N°	Gene Symbols	Description	Median expression value n=4		Mean Fold Change n=4	GO terms BP	Transcription factor binding sites in promotor
			Control	DHA	DHA/Control		
ens|ENSBTAT00000026534	**GSN**	gelsolin	7.6	2.6	0.33	protein binding	**HNF-4**alpha2 HNF-4alpha1 Sp1 NF-AT2 Evi-1 NF-AT NF-AT1 NF-AT3 NF-AT4 GATA-1
ens|ENSBTAT00000043699	**LLGL1**	lethal giant larvae homolog 1 (Drosophila)	9.2	3.8	0.42	cortical actin cytoskeleto organisation	nd
ens|ENSBTAT00000063557	MTA1	metastasis associated 1	10.0	4.1	0.43	regulation of transcription, signal transduction	RFX1 GATA-3 Tal-1 E47 AML1a FOXD1 HOXA3 En-1 Pax-5
ens|ENSBTAT00000022366	GRO1	chemokine (C-X-C motif) ligand 1 (melanoma growth stimulating activity,	2.5	1.4	0.50	protein binding, chemokine activity,	nd
ens|ENSBTAT00000016827	NDST2	N-deacetylase/N-sulfotransferase (heparan glucosaminyl) 2	4.1	2.1	0.53	hydrolase activity, sulfotransferase activity	GR-alpha Nkx3-1v4 AP-2alpha AP-2alphaA AP-2beta AP-2gamma GR Nkx3-1v3 Nkx2-5 Egr-4
ens|ENSBTAT00000045994	CXCL3	chemokine (C-X-C motif) ligand 3	3.0	1.5	0.54	inflammatory & immune responce	ARP-1 Max1 Ik-1 HNF-3beta STAT1 STAT1alpha STAT1beta STAT2 STAT3 STAT4
ens|ENSBTAT00000001619	**RBM14**	RNA binding motif protein 14	18.2	10.1	0.55	RNA splicing modulator, RNA & protein binding	CUTL1 AREB6 HOXA5 c-Myb NF-1 NF-1/L CBF(2) NF-Y FAC1 XBP-1
ens|ENSBTAT00000057477	**MTMR3**	myotubularin related protein 3	6.2	3.4	0.56	protein tyrosine phosphatase activity and phosphatidylinositol-3-phosphatase activity	
ens|ENSBTAT00000057307	**PRR5**	proline rich 5 (renal)	2.5	1.4	0.56	cell cycle, protein phosphorylation	**PPAR-gamma2 PPAR-gamma1** E47 HEN1 **NF-kappaB NF-kappaB1**
ens|ENSBTAT00000032442	NBAS	^{2}	1.6	0.9	0.59	protein binding	POU3F2 GATA-1 USF-1 USF-1:USF-2 USF1 USF2 Nkx3-1 Nkx3-1v1 Nkx3-1v2 Nkx3-1v3
ens|ENSBTAT00000027875	**HSD17B8**	hydroxysteroid (17-beta) dehydrogenase 8	4.5	2.7	0.59	FA, estrogen, androgen biosynthetic processes, protein I	Sp1
ens|ENSBTAT00000019167	C5H12orf23	chromosome 5 open reading frame, alias TMEM263 Transmembrane Pro	19.1	11.2	0.60	transmembrane protein	nd
ens|ENSBTAT00000064986	UBFD1	ubiquitin domain-containing protein	42.0	25.3	0.60	RNA/protein binding	Nkx2-5 p53 Pax-4a NF-1 POU3F1 NF-1/L Pax-2a Pax-2b Pax-2 Elk-1
ens|ENSBTAT00000005231	PMM1	phosphomannomutase 1	19.4	12.2	0.60	catalytic activity, protein binding	ATF6 XBP-1 AML1a c-Myb ATF-2 MZF-1 STAT5B STAT5A
ens|ENSBTAT00000054137	ANXA11	annexin A11	2.7	1.6	0.60	phagocytosis, cell cycle, cytokinesis	IRF-1 Bach2 RSRFC4 Arnt GR-alpha GR GR-beta
ens|ENSBTAT00000065279	ZNF281	zinc finger protein 281	15.0	8.9	0.60	DNA binding transcription factor activity a	Nkx2-5 Lmo2 Pax-4a c-Myc Max1 FOXJ2 FOXJ2(longisoform) STAT5A C/EBPalpha
ens|ENSBTAT00000011507	MAGED1	melanoma antigen family D, 1	75.0	45.1	0.61	regulation of transcription and apoptosis	
ens|ENSBTAT00000030078	**DPF2**	D4, zinc and double PHD fingers family 2	29.1	17.2	0.61	regulation of transcription, apoptosis	SEF-1(1) LCR-F1 Sp1 Elk-1 STAT5A Sox5 AML1a NRF-2 IRF-2
ens|ENSBTAT00000024373	**PPP2R2A**	protein phosphatase 2, regulatory subunit B, alpha	25.3	15.3	0.61		**SREBP-1b SREBP-1a SREBP-1c** gr-1 ATF-2 GATA-3 FOXD3 MZF-1 FOXO1a FOXO1
ens|ENSBTAT00000005555	**ARPC1A**	actin related protein 2/3 complex, subunit 1A, 41kDa	8.6	5.3	0.62	Actin filament binding, protein binding	Msx-1 **SREBP-1c SREBP-1b SREBP-1a** AhR Arnt AREB6 POU2F1a POU2F1
ens|ENSBTAT00000021109	UBE2J2	ubiquitin-conjugating enzyme E2, J2	16.8	10.1	0.62	ubiquitin-protein transferase activity	Tal-1beta ITF-2 ATF6 Roaz N-Myc AhR NF-1 LCR-F1 CP2
ens|ENSBTAT00000019970	VCP	valosin containing protein (*secreted)*	116.7	85.3	0.63	**ATP binding,** receptor binding, protein	**NF-kappaB1** delta**CREB CREB POU3F2 POU3F2**(N-Oct-5b) POU3F2(N-Oct-5a) RREB-1 Pax-5 STAT3 GR
ens|ENSBTAT00000000786	ANKLE2	ankyrin repeat and LEM domain containing 2	3.5	2.1	0.63	Mitosis, protein binding,regulator of PP2A	FOXI1 MIF-1 HFH-3 FOXL1 NRSFform2 NRSFform1 NF-AT Ik-3 HFH-1 NF-AT1
ens|ENSBTAT00000027662	PCBP2	poly(rC) binding protein 2	90.7	56.9	0.63	mRNA splicing, gene expression	HFH-1 RORalpha1 HSF1(long) E47 Tal-1beta HSF1short MIF-1 TBP Bach2 Elk-1
ens|ENSBTAT00000007881	STAP2	signal transducing adaptor family member 2	3.3	2.1	0.64	**phospholipid binding**	STAT3 **NF-kappaB1 NF-kappaB** STAT5A USF2
ens|ENSBTAT00000037020	UBE2F	ubiquitin-conjugating enzyme E2F (putative)	4.6	2.8	0.64	NEDD8 transferase activity	FOXD1 GATA-6 AML1a HNF-3beta RORalpha2 TBP TFIID NF-Y CBF(2) LUN-1
ens|ENSBTAT00000024092	**AHCY**	adenosylhomocysteinase	193.3	113.2	0.64	Metabolism, methylation, protein binding	POU2F1a POU2F1 AP-4 C/EBPalpha COMP1 aMEF-2 MEF-2A GATA-3
ens|ENSBTAT00000003167	AES	amino-terminal enhancer of split	2.5	1.6	0.64	Transcription corepressor activity, protein binding	nd
ens|ENSBTAT00000000941	GUSB	glucuronidase, beta	1.6	1.0	0.64	carbohydrate metabolism	TBP Sp1 AML1a SEF-1(1) Ik-2
ens|ENSBTAT00000032269	QPCTL	glutaminyl-peptide cyclotransferase-like	6.8	4.2	0.65	peptidase activity and glutaminyl-peptide cyclotransfera	AREB6 Max1 c-Myc AP-2gamma **PPAR-gamma2 PPAR-gamma1** GATA-1 AML1a
ens|ENSBTAT00000029701	ULK2	Unc-51 Like Autophagy Activating Kinase 2	1.3	1.1	0.65	catalytic & protein kinase activity	Elk-1 ER-alpha NF-AT NF-AT4 NF-AT3 NF-AT2 NF-AT1 AP-2alpha
ens|ENSBTAT00000045996	GCDH	glutaryl-CoA dehydrogenase	2.6	1.8	0.66	**Fatty acid oxidation**, aa metabolism,	nd
ens|ENSBTAT00000052059	HNRNPU	heterogeneous nuclear ribonucleoprotein U (scaffold attachment factor	30.9	20.8	0.66	DNA, RNA binding	deltaCREB **CREB**
ens|ENSBTAT00000009627	**PI4KB**	phosphatidylinositol 4-kinase, catalytic, beta	6.1	4.1	0.66	ATP binding, tprotein binding, phosphotransferase activi	Pax-5 ATF-2 CP2 c-Myc Max1 AP-2gamma IRF-7A LCR-F1 C/EBPbeta HFH-3
ens|ENSBTAT00000025144	ATP6V0B	ATPase, H+ transporting, lysosomal 21kDa, V0 subunit b	5.9	3.7	0.66	Membrane component, endosome	E2F-3a FAC1 c-Ets-1 E2F E2F-1 E2F-2 E2F-4 E2F-5 GATA-2 Ik-2
ens|ENSBTAT00000023493	DHRS4	dehydrogenase/reductase (SDR family) member 4 (DHRS4).	21.7	13.5	0.66	steroid metabolism	IRF-7A Nkx2-5 C/EBPalpha NF-E2 NF-E2p45 Oct-B1 oct-B2 oct-B3 POU2F1 POU2F1a
ens|ENSBTAT00000011020	FBXL20	F-box/LRR-repeat protein 20	1.3	0.9	0.66	protein binding	AP-1 c-Jun ATF-2 STAT3 AREB6 SRY C/EBPalpha Arnt Cdc5
ens|ENSBTAT00000052354	SULT1A1	sulfotransferase family, cytosolic, 1A, phenol-preferring, member 1	2.5	1.5	0.67	protein binding, steroid sulfotransferase activity	STAT3
ens|ENSBTAT00000013093	KIAA1737	KIAA1737 ortholog alias CIPC CLOCK-Interacting Pacemaker	4.6	3.1	0.67	transcription, rhytmic process	
ens|ENSBTAT00000018863	CTNNA1	catenin (cadherin-associated protein), alpha 1	32.0	21.5	0.67	ovarian foll development, actin filament organisation, ce	IRF-1 GR-alpha GR POU3F2 HOXA9 HOXA9B Meis-1 Meis-1a POU3F2(N-Oct-5a)
ens|ENSBTAT00000025346	COMMD9	COMM domain containing 9	21.8	15.0	0.67	regulation of transcription, sodium ion transport	AREB6 FOXO3b FOXO3a FOXO3 Hand1 E47 GR GR-alpha GR-beta CUTL1
ens|ENSBTAT00000005592	SGK1	serum/glucocorticoid regulated kinase 1	6.6	4.4	0.67		
ens|ENSBTAT00000039630	ZNF408	zinc finger protein 408	1.5	0.9	0.68		
ens|ENSBTAT00000052666	SLC39A7	solute carrier family 39 (zinc transporter)	2.1	1.5	0.68		
ens|ENSBTAT00000028761	TPGS2	tubulin polyglutamylase complex subunit 2	3.2	2.3	0.69		
ens|ENSBTAT00000045436	C2	complement component 2	1.1	0.8	0.70	catalic activity, proteolisis, responce to nutrient, comple	AP-1 IRF-1 C/EBPbeta MyoD c-Jun
ens|ENSBTAT00000044257	TUBB2B	Tubulin beta-2B chain	117.2	82.8	0.70		
ens|ENSBTAT00000063075	FAM174A	family with sequence similarity 174, member A	3.3	2.4	0.70	integral component of membrane	Nkx6-1 POU2F1a POU2F1 c-Ets-1 HSF1short HSF1(long) Pax-4a AREB6 NRF-2 Pbx1a
ens|ENSBTAT00000035886	GNL1	guanine nucleotide binding protein-like 1	1.3	0.9	0.71	GTPase activity, signal transduction	AREB6 C/EBPalpha Pax-5 **SREBP-1c SREBP-1b SREBP-1a** p53 MZF-1
ens|ENSBTAT00000002327	SIRT2	sirtuin 2	55.1	39.1	0.71		
ens|ENSBTAT00000044104	MYBPC3	myosin binding protein C, cardiac	1.4	1.0	0.71	ATPase activator, protein binding	AML1a ER-alpha HOXA5 **NF-kappaB1 NF-kappaB** Egr-1 AP-1 **SREBP-1a SREBP-1b**
ens|ENSBTAT00000012252	PPP1R35	Protein phosphatase 1 regulatory subunit 35	155.6	112.1	0.71	phosphatase binding, protein phosphatase inhibitor activity.	
ens|ENSBTAT00000064981	SDHA	succinate dehydrogenase complex, subunit A, flavoprotein	141.9	105.1	0.71		
ens|ENSBTAT00000002549	MRPS27	mitochondrial ribosomal protein S27	39.8	28.2	0.71	protein binding	**NF-kappaB NF-kappaB1 NF-kappaB2** Evi-1 Gfi-1 RORalpha1 NRSFform2 NRSFform1 HTF
ens|ENSBTAT00000024072	QPRT	quinolinate phosphoribosyltransferase	28.4	19.2	0.71		
ens|ENSBTAT00000010154	MOCS3	molybdenum cofactor synthesis 3	3.2	2.2	0.72	metabolism, protein binding	STAT5A HOXA5 NRF-2 c-Myc Max1 C/EBPalpha Roaz COMP1
ens|ENSBTAT00000015353	SURF6	surfeit 6	21.3	16.0	0.72		
ens|ENSBTAT00000008501	TSEN34	tRNA splicing endonuclease 34 homolog (S. cerevisiae)	73.3	52.5	0.73		
ens|ENSBTAT00000004645	DDX19A	DEAD (Asp-Glu-Ala-As) box polypeptide 19A	5.2	4.1	0.73	RNA binding, mRNA export from the nucleus	c-Ets-1 FOXO4 SEF-1(1) C/EBPbeta Meis-1a Meis-1 Meis-1b Max c-Myc ATF-2
ens|ENSBTAT00000011732	TMEM98	transmembrane protein 98	60.7	43.4	0.73		
ens|ENSBTAT00000013522	DHRS1	dehydrogenase/reductase (SDR family) member 1	20.6	14.6	0.73	oxidation-reduction process, protein binding	HNF-4alpha1 Roaz c-Ets-1 GR GR-alpha
ens|ENSBTAT00000015026	CCDC12	coiled-coil domain containing 12	13.9	10.7	0.73	protein binding	**NF-kappaB1 NF-kappa** B NF-1 CP2 c-Ets-1 ZID Nkx2-5 Lmo2**CRE** B HOXA
ens|ENSBTAT00000012411	PMF1	polyamine-modulated factor 1	2.0	1.5	0.73	cell cycle, mitosis	TBP GR-alpha GR GR-beta c-Jun ATF-2 YY1 HOXA5

ens|ENSBTAT00000064376	TPRN	taperin	1.8	1.3	0.73		
ens|ENSBTAT00000020635	CHCHD4	coiled-coil-helix-coiled-coil-helix domain containing 4	7.4	5.4	0.73	protein trageting to mitochondrion, oxidative stress	nd
ens|ENSBTAT00000015716	*EEF1G*	eukaryotic translation elongation factor 1 gamma	183.2	123.4	0.73	translation elongation factor activity, protein binding	nd
ens|ENSBTAT00000004365	SMG8	smg-8 homolog, nonsense mediated mRNA decay factor (C. elegans)	1.5	1.2	0.74		
ens|ENSBTAT00000008028	*EXOC8*	exocyst complex component 8	9.7	7.3	0.74		E2F E2F-4 E2F-3a E2F-2 E2F-1 E2F-5 c-Myb GATA-1 HOXA9 HOXA9B
ens|ENSBTAT00000014415	DCP1A	Decapping MRNA 1A	26.1	19.3	0.74	nuclear-transcribed mRNA catabolic process, gene expre	nd
ens|ENSBTAT00000036467	IL11RA	interleukin 11 receptor, alpha	8.5	6.5	0.74	signal transduction, developmental process, embryo imp	AP-1 p53
ens|ENSBTAT00000017473	**DDHD2**	DDHD domain containing 2	10.5	7.6	0.75	**lipid catabolic process**, protein binding,	Sp1 Pax-5 POU2F1a POU2F1 CUTL1 AML1a TBP Cart-1
ens|ENSBTAT00000006381	PLEKHO1	pleckstrin homology domain containing, family O member 1	42.5	32.2	0.75	protein binding	AP-1 c-Jun ATF-2 Evi-1 MyoD COMP1 E47 Tal-1 STAT3
ens|ENSBTAT00000024621	PQBP1	polyglutamine binding protein 1	1.9	1.3	0.76		
ens|ENSBTAT00000018998	RHOC	ras homolog gene family, member C	88.4	65.0	0.76		
ens|ENSBTAT00000014853	FTH1	ferritin, heavy polypeptide 1	538.7	408.2	0.76	iron ion transport, receptor mediated endocytosis, ferro	STAT1 RP58 Nkx2-5 Pax-5 AP-1 GATA-1 HSF2 Gfi-1
ens|ENSBTAT00000030501	TMEM186	transmembrane protein 186	7.2	5.4	0.77		
ens|ENSBTAT00000063693	PHF23	PHD finger protein 23	1.6	1.2	0.77	protein binding	AP-2apha COUP COUP-TF COUP-TF1 HNF-4alpha1 HNF-4alpha2
ens|ENSBTAT00000049671	DONSON	downstream neighbor of SON	2.7	1.9	0.77	multicellulr organism development	nd
ens|ENSBTAT00000004027	SART1	squamous cell carcinoma antigen recognized by T cells	1.0	0.9	0.77		
ens|ENSBTAT00000027314	ACP1	acid phosphatase 1, soluble	41.2	32.1	0.78	Acid phospahatase activity, protein binding	nd
ens|ENSBTAT00000012142	TOM40B	translocase of outer mitochondrial membrane 40 homolog (yeast)-like	3.7	2.8	0.78		
ens|ENSBTAT00000007195	BIN3	bridging integrator 3	38.1	30.1	0.78	Protein binding, Cytoskeletal adaptor activity, cytokinesi	Nkx3-1v4 Nkx3-1v3 Nkx3-1v2 Nkx3-1v1
ens|ENSBTAT00000025246	POLR1C	polymerase (RNA) I polypeptide C	19.2	15.0	0.78	transcription regulation	SRF(504AA) SRF CUTL1 RP58 STAT3 RREB-1 FOXD1 c-Myb GR GR-beta
ens|ENSBTAT00000025026	SNX3	sorting nexin 3	57.2	45.2	0.79		
ens|ENSBTAT00000028329	*GALNT11*	polypeptide N-acetylgalactosaminyltransferase 11	4.9	4.0	0.79	glycosylation, Notch signaling, metabolic process	nd
ens|ENSBTAT00000022398	MTO1	mitochondrial translation optimization 1 homolog (S. cerevisiae)	2.2	1.8	0.80	tRNA processing, oxidation-reduction process	
ens|ENSBTAT00000043714	NDUFV3	NADH dehydrogenase (ubiquinone) flavoprotein 3	1.2	1.0	0.80	mitochondria electron transport, cellule metabolism	**PPAR-gamma2 PPAR-gamma1** HEN1 Hand1 E47 POU2F1a POU2F1 Zic3 c-Jun ATF-2
ens|ENSBTAT00000023118	C3H1ORF50	chromosome 3 open reading frame, human C1orf50	14.6	12.3	0.81	catalic activity, protein binding	AREB6 COMP1 AML1a Olf-1 **PPAR-alpha** POU2F1a POU2F1 c-Myc p300 Max
ens|ENSBTAT00000035195	FAN1	FANCD2/FANCI-associated nuclease 1	1.9	1.4	0.81	DNA catabolic process, endonucleolytic	nd
ens|ENSBTAT00000017580	SLC25A4	solute carrier family 25 (mitochondrial carrier; adenine nucleotide transl	6.6	5.5	0.82		
ens|ENSBTAT00000023705	RBMS2	RNA binding motif, single stranded interacting protein 2	2.8	2.3	0.83		
ens|ENSBTAT00000020308	SGSH	N-sulfoglucosamine sulfohydrolase	1.3	1.0	0.84		
ens|ENSBTAT00000059168	SNORA38	Small nucleolar RNA SNORA38	1.7	2.1	1.22		
ens|ENSBTAT00000001831	MTF2	metal response element binding transcription factor 2 .	28.6	36.0	1.25	chromatin modification, regulation of transcription	MZF-1 p300 Pax-4a IRF-7A HSF1 **CREB** GCNF GCNF-1
ens|ENSBTAT00000012144	ETS2	v-ets erythroblastosis virus E26 oncogene homolog 2 (avian)	3.7	4.7	1.25		
ens|ENSBTAT00000025941	NR3C1	nuclear receptor subfamily 3, group C, member 1 (glucocorticoid recepto	1.3	1.8	1.25	steroid hormone receptor activity, transcription factor and regulation of transcription (GRE-binding)	
ens|ENSBTAT00000013500	CPSF2	cleavage and polyadenylation specific factor 2	6.5	8.1	1.26	RNA, binding, protein binding,	nd
ens|ENSBTAT00000043322	**EIF2S1**	eukaryotic translation initiation factor 2, subunit 1 alpha	66.0	91.0	1.26	nucleic acid binding, translation initiation	nd
ens|ENSBTAT00000001533	**TLE6**	transducin-like enhancer of split 6 (E(sp1) homolog, Drosophila)	279.7	358.9	1.29	regulation of transcription, neuron differentiation	c-Jun ATF-2 AP-1 p53 c-Ets-1 USF-1 USF-1:USF-2 USF1 USF2 LUN-1
ens|ENSBTAT00000026549	DDHD1	DDHD domain containing 1 (Phosphatidic Acid-Preferring Phospholipase	1.9	2.6	1.31	lipid catabolic process, metal ion binding	Egr-4 Arnt AhR c-Myb c-Ets-1 Tal-1beta ITF-2 FOXJ2 FOXJ2(longisoform) Pax-2b
ens|ENSBTAT00000004528	MAGOH	mago-nashi homolog, proliferation-associated (Drosophila)	35.7	45.0	1.32	gene expression, RNA transport, RNA binding	SRF(504AA) SRF CUTL1 FOXO3b FOXO3a FOXO3 Hand1 E47 XBP-1 COMP1
ens|ENSBTAT00000060283	U6atac	U6atac minor spliceosomal RNA	1.1	1.6	1.35		
ens|ENSBTAT00000059327	SNORA3	Small nucleolar RNA SNORA3/SNORA45 family	1.0	1.3	1.38		
ens|ENSBTAT00000065876	**Metazoa_SRP**	Metazoan signal recognition particle RNA	631.1	985.6	1.62	nd	
ens|ENSBTAT00000060875	**U4**	U4 spliceosomal RNA	924.9	1313.9	1.66	RNA processing	
ens|ENSBTAT00000064588	SNORA18	Small nucleolar RNA SNORA18	136.8	213.2	1.70	RNA processing	
ens|ENSBTAT00000059655	SNORA73	Small nucleolar RNA SNORA73 family	49.0	77.7	1.71	RNA processing	
ens|ENSBTAT00000059765	SNORA42	Small nucleolar RNA SNORA42/SNORA80 family	21.4	39.6	1.73	RNA processing	
ens|ENSBTAT00000060085	SNORA11	Small nucleolar RNA SNORA11	7.5	13.6	1.79	RNA processing	
ens|ENSBTAT00000060369	**SNORD16**	Small nucleolar RNA SNORD16	121.4	221.8	1.83	RNA processing	
ens|ENSBTAT00000060503	SNORA61	Small nucleolar RNA SNORA61	10.1	18.6	1.83	RNA processing	
ens|ENSBTAT00000059944	U6	U6 spliceosomal RNA	1.0	1.5	1.86	RNA processing	
ens|ENSBTAT00000059715	**U11**	U11 spliceosomal RNA	18.1	40.2	1.93	RNA processing	
ens|ENSBTAT00000059928	SNORD96	Small nucleolar RNA SNORD96 family	10.8	20.8	1.93	RNA processing	
ens|ENSBTAT00000059490	SNORA2	Small nucleolar RNA SNORA2/SNORA34 family	1.1	1.8	1.94	RNA processing	
ens|ENSBTAT00000059757	SNORA43	Small nucleolar RNA SNORA43	21.6	43.5	1.96	RNA processing	
ens|ENSBTAT00000063045	SNORA63	Small nucleolar RNA SNORA63	1.9	2.4	1.96	RNA processing	
ens|ENSBTAT00000059474	**SNORA17**	Small nucleolar RNA SNORA17	89.6	190.7	2.06	RNA processing	Ik-2 c-Myc AP-2gamma SRF(504AA) **SRF Max deltaCREB** C/EBPalpha CHOP-10 AML1a
ens|ENSBTAT00000059126	SNORD14	Small nucleolar RNA SNORD14	7.7	17.0	2.07	RNA processing	
ens|ENSBTAT00000060880	SNORA35	Small nucleolar RNA SNORA35	11.5	23.9	2.10	RNA processing	HEN1 HSF2 p53 AML1a Arnt POU6F1(c2) GATA-3 AREB6 YY1 LCR-F1
ens|ENSBTAT00000051513	**U1**	U1 spliceosomal RNA	232.2	400.7	2.10	RNA processing	
ens|ENSBTAT00000060209	SNORA29	Small nucleolar RNA SNORA29	10.3	22.5	2.16	RNA processing	p53 Egr-2 IRF-1 TBP Lmo2 Pax-4a SRY USF-1:USF-2 USF-1
ens|ENSBTAT00000059571	**snoU6-53**	Small nucleolar RNA U6-53/MBII-28 :SNORD8	36.7	82.8	2.27	RNA processing	TBP TFIID E2F-4 E2F-3a E2F-2 E2F-1 E2F
ens|ENSBTAT00000059978	**SNORA16**	Small nucleolar RNA SNORA16B/SNORA16A family	13.5	31.9	2.34	RNA processing	SRY Nkx6-1 SREBP-1c **SREBP-1b SREBP-1a** AML1a TBP FOXL1 AREB6 C/EBPalpha
ens|ENSBTAT00000059863	SNORA44	Small nucleolar RNA SNORA44	5.6	13.2	2.39	RNA processing	AREB6 TGIF AML1a CP2 SEF-1(1) Meis-1b Meis-1a Meis-1 STAT5A GR-beta
ens|ENSBTAT00000060351	**SNORA21**	Small nucleolar RNA H/ACA Box 21	10.2	24.1	2.43	RNA processing	**deltaCREB CREB** RFX1 AhR Arnt AREB6 Evi-1 CBF(2)
ens|ENSBTAT00000059793	**SNORD100**	Small nucleolar RNA C/D Box 100)	48.6	163.0	3.44	RNA processing	**PPAR-gamma1 PPAR-gamma2** Brachyury YY1 **FOXO3 FOXO3a FOXO3b c-**Myb GR-alpha GR
