# Supplementary material for: Docosahexaenoic acid mechanisms of action on the bovine oocyte-cumulus complex
Source: J Ovarian Res. 2017 Nov 9;10:74. doi: 10.1186/s13048-017-0370-z (PMC5679375; doi:10.1186/s13048-017-0370-z)
Supplement: Supplementary file 2 — Cumulus gene expression during maturation in absence or presence of DHA 1 μM. Relative expression is expressed relatively to the geometric mean of 3 housekeeping genes (RPL19, RPS9 and GAPDH) and presented as mean ± sem. (PDF 1677 kb) [file 13048_2017_370_MOESM2_ESM.pdf]

Additional file 2: Table S2 : Cumulus gene expression during maturation in absence or presence of DHA 1µM. Relative expression is expressed relatively to the geometric mean of 3 housekeeping genes (*RPL19*, *RPS9* and *GAPDH*) and presented as mean ± sem.

	Control				DHA 1µM			ANOVA
	0h	4h	10h	24h	4h	10h	24h	p-value
AHCY	0.0669 ± 0.0041 a	0.0514 ± 0.0034 bc	0.042 ± 0.0033 c	0.0473 ± 0.0079 abc	0.0605 ± 0.0037 ab	0.0523 ± 0.0038 bc	0.0428 ± 0.0063 c	0.0011
DHRS1	0.0298 ± 0.0023 a	0.0196 ± 0.0018 bc	0.0192 ± 0.0021 bc	0.0296 ± 0.0024 ad	0.0205 ± 0.0021 bc	0.0158 ± 0.0034 bc	0.0293 ± 0.0053 ac	0.0011
EEF1G	0.1626 ± 0.0129 a	0.1238 ± 0.0081 bc	0.1442 ± 0.0091 ab	0.1014 ± 0.0123 c	0.1377 ± 0.0118 abc	0.1558 ± 0.0091 ab	0.12 ± 0.0218 abc	0.0238
EIF2S1	0.2798 ± 0.0431 ab	0.4254 ± 0.0301 bc	0.4884 ± 0.0352 b	0.378 ± 0.0438 acd	0.4493 ± 0.023 bc	0.5096 ± 0.0305 bd	0.2759 ± 0.029 a	<0.0001
GLUT1	4616 ± 1011 a	4063 ± 947 a	1010 ± 145 bc	393 ± 98 c	4845 ± 974 a	1184 ± 261 b	648 ± 148 bc	0.0004
GPX1	0.4941 ± 0.0479 a	0.3185 ± 0.0226 b	0.0664 ± 0.0039 d	0.2418 ± 0.011 c	0.3361 ± 0.0213 ab	0.0654 ± 0.0049 d	0.2082 ± 0.0185 c	<0.0001
GPX4	0.7011 ± 0.1109 ab	0.9207 ± 0.044 a	0.9429 ± 0.0688 a	0.6825 ± 0.0536 b	0.9461 ± 0.0557 a	0.905 ± 0.0619 a	0.4335 ± 0.0749 b	0.0003
GSN	0.124 ± 0.0184 a	0.1519 ± 0.0212 a	0.5016 ± 0.113 b	0.4397 ± 0.0685 b	0.1155 ± 0.0184 a	0.389 ± 0.0365 b	0.4567 ± 0.0798 b	<0.0001
Met_SRP	40.4 ± 4.1 a	53.2 ± 4.9 ac	65.5 ± 6 bcd	163.1 ± 46.9 cd	48.7 ± 6 ab	67.2 ± 6.8 cd	143.3 ± 47 d	0.0004
MTMR3	0.0053 ± 0.0007 a	0.0071 ± 0.0006 ab	0.0112 ± 0.0014 bc	0.0088 ± 0.0014 bc	0.0079 ± 0.0007 b	0.0131 ± 0.0016 c	0.0073 ± 0.0011 ab	0.0001
SIRT2	0.0494 ± 0.0016 b	0.0474 ± 0.0034 a	0.1006 ± 0.0067 ed	0.0751 ± 0.0088 bd	0.0446 ± 0.0035 ac	0.1031 ± 0.0057 e	0.0793 ± 0.014 abce	<0.0001
SNORA16	1.256 ± 0.1239 a	3.8289 ± 0.687 bc	9.4258 ± 1.5634 d	9.4219 ± 3.4371 cd	3.277 ± 0.6812 ab	10.4333 ± 1.8371 d	9.6781 ± 3.345 bd	0.0002
SNORA17	0.6279 ± 0.0889 a	1.6848 ± 0.3016 bc	3.3456 ± 0.4413 d	2.891 ± 0.8709 cd	1.1722 ± 0.2063 ab	3.2206 ± 0.4487 d	1.9756 ± 0.3586 bd	<0.0001
SNOU6_53	0.1396 ± 0.0093 a	0.4252 ± 0.0753 b	0.8673 ± 0.1058 cd	0.6046 ± 0.1384 bd	0.2881 ± 0.0533 ab	1.1279 ± 0.1945 d	0.4707 ± 0.0438 bc	<0.0001
U1	0.0224 ± 0.0035 bc	0.0107 ± 0.0014 ab	0.0259 ± 0.0033 c	0.0834 ± 0.0371 c	0.0097 ± 0.0013 a	0.029 ± 0.0038 bc	0.0571 ± 0.0195 c	0.0007
VCP	0.126 ± 0.0167 ac	0.1335 ± 0.0101 ab	0.1524 ± 0.0121 a	0.1005 ± 0.0151 bc	0.1522 ± 0.0173 ab	0.1769 ± 0.015 a	0.0908 ± 0.0141 c	0.0107

Different letters on the same row indicate significant differences between timepoints and/or conditions $p < 0.05$
