# Supplementary material for: Docosahexaenoic acid mechanisms of action on the bovine oocyte-cumulus complex
Source: J Ovarian Res. 2017 Nov 9;10:74. doi: 10.1186/s13048-017-0370-z (PMC5679375; doi:10.1186/s13048-017-0370-z)
Supplement: Supplementary file 3 — Lipids detected by MALDI MS in bovine cumulus cells (CC) and oocytes (OO), in positive (+) et negative (−) modes. (PDF 609 kb) [file 13048_2017_370_MOESM3_ESM.pdf]

Additional file 3: Table S3. Lipids detected by MALDI MS in bovine cumulus cells (CC) and oocytes (OO), in positive (+) et negative (-) modes.

OO (+) m/z (406)	OO(-) m/z (294)	CC (+) m/z (324)	CC (-) m/z (374)
200.15	208.16	203.19	205.17
202.42	209.16	206.19	207.24
203.27	210.17	212.13	208.20
206.27	211.16	217.21	209.19
212.20	213.05	219.19	211.21
215.18	213.69	227.19	212.21
216.27	214.23	233.21	213.07
217.47	217.23	239.21	213.67
219.28	218.25	243.17	216.24
227.28	227.21	245.19	217.27
231.29	231.17	253.16	218.29
233.30	237.28	255.17	225.16
238.36	243.25	257.21	227.14
239.32	245.24	261.19	229.10
241.28	248.11	264.22	231.22
242.27	249.13	267.18	233.20
243.28	251.13	268.18	235.29
245.28	253.13	269.18	236.30
251.22	259.21	271.22	241.19
253.28	260.25	274.36	242.26
255.29	264.20	279.19	243.28
256.29	265.21	280.21	245.26
257.29	269.25	281.21	247.24
260.17	271.19	283.18	248.01
261.29	272.21	285.19	249.19
264.46	279.23	286.19	250.63
265.29	281.40	287.20	251.64
267.27	283.22	295.16	255.39
268.29	284.23	297.22	257.26
269.29	285.24	299.21	258.24
271.31	286.17	301.19	259.28
274.45	287.18	302.20	260.30
275.30	291.04	303.20	261.30
276.13	293.35	307.22	263.22
279.30	297.29	309.17	264.23
280.31	299.22	312.45	265.24
281.33	300.22	313.20	269.26
284.33	301.23	319.17	270.21
287.30	303.27	321.20	271.22
290.32	309.34	325.13	272.25
295.27	311.31	326.43	273.24
297.32	314.20	329.18	277.11
299.29	315.22	332.42	278.54
301.21	317.23	335.49	279.22
303.29	319.22	337.22	281.40
307.32	321.35	339.41	283.27
309.28	322.15	340.34	284.28
311.29	323.21	341.42	285.27
313.28	324.22	342.17	286.23
317.27	325.30	343.17	287.23
318.29	329.22	351.22	291.16
319.32	330.20	353.20	293.35
320.31	331.22	355.25	297.35
321.31	333.21	356.25	299.27
324.31	335.38	357.07	300.28
325.02	337.40	358.06	301.25
326.56	339.32	363.25	303.30
327.29	341.25	365.23	305.33
329.28	345.32	368.49	309.34
331.27	346.14	369.47	311.36
332.51	347.82	371.23	313.25
333.11	349.20	372.40	314.25
335.12	350.22	373.25	315.25
337.28	351.21	376.16	317.24
339.47	352.22	377.18	319.25
340.55	353.32	379.20	321.40
341.32	357.14	381.19	322.25
342.27	361.18	383.20	323.26
343.27	362.14	385.24	324.27
347.28	364.16	387.23	325.27
349.30	365.17	388.46	329.25
351.28	367.32	391.20	330.25
353.28	368.27	393.20	331.26
355.28	369.23	395.21	333.23
356.19	371.27	397.17	335.35
357.21	372.68	401.19	337.28
358.18	375.23	402.30	339.37
361.22	376.11	405.18	341.29
363.29	377.20	407.18	345.25
365.27	379.21	411.17	346.23
368.59	383.19	413.19	347.83
371.28	385.14	415.21	349.26
372.41	388.44	417.19	350.25
373.31	391.22	419.19	351.24
375.28	392.21	421.21	353.23
377.27	395.18	423.15	354.24
379.28	397.30	425.15	355.24
381.24	399.17	428.30	357.23
383.25	401.13	430.44	358.04
385.32	403.14	433.18	359.23
387.25	405.18	435.19	361.19
388.56	407.21	437.20	363.20
389.30	409.21	439.21	364.22
390.29	411.22	441.22	365.20
391.29	413.22	443.20	366.17
392.29	415.20	445.19	367.23
393.28	416.07	447.20	369.25
395.29	417.05	449.18	371.25
396.31	419.18	452.54	372.67
397.31	421.15	453.21	375.22
398.87	431.22	455.18	376.16
401.29	432.21	457.19	377.24

OO (+) m/z (406)	OO(-) m/z (294)	CC (+) m/z (324)	CC (-) m/z (374)
402.46	433.19	459.17	379.07
403.26	435.20	461.18	381.24
405.24	437.21	463.19	383.20
407.29	439.21	469.18	385.11
409.29	441.16	471.19	388.20
411.28	443.18	473.20	391.26
413.26	445.18	475.18	392.27
415.30	447.20	477.17	395.20
417.28	449.19	479.20	397.22
419.28	451.20	481.19	399.23
421.30	452.25	485.19	401.17
423.24	453.24	487.20	403.19
427.28	454.07	489.20	405.23
428.34	455.17	491.18	407.24
429.30	459.18	493.14	408.24
430.75	461.11	495.17	409.21
431.31	462.12	496.40	411.21
433.30	463.16	499.20	413.23
435.29	465.37	501.18	415.22
437.30	471.15	502.21	416.10
439.31	473.17	509.16	417.12
441.31	475.17	511.11	421.22
443.28	477.16	513.13	423.21
445.30	478.34	515.18	425.20
447.32	483.22	517.19	427.22
449.28	485.53	520.57	429.19
452.64	487.17	522.42	431.24
453.30	488.15	524.42	433.21
455.31	489.15	527.23	434.26
457.30	490.13	530.24	435.24
459.29	491.11	533.18	437.26
461.27	492.11	538.18	439.24
463.30	493.12	539.13	441.23
466.06	497.50	541.20	445.20
467.30	499.26	543.19	446.20
468.46	500.35	544.39	447.24
469.29	503.33	545.21	449.22
470.35	505.17	548.58	451.23
471.30	507.11	549.18	452.30
473.31	508.09	550.52	453.27
475.30	509.09	553.16	454.16
477.29	513.55	555.17	455.22
478.28	515.18	557.16	459.20
479.26	517.15	561.17	460.17
480.44	519.47	564.97	461.19
481.26	521.14	567.18	462.17
485.26	524.35	569.18	463.19
487.27	526.35	570.38	465.40
489.29	528.36	571.14	469.19
491.28	529.19	572.37	471.20
493.25	533.15	574.18	472.20
494.45	535.15	575.53	473.19
495.80	537.14	577.56	475.21
496.45	539.19	582.16	477.21
499.27	541.16	588.26	478.39
501.23	555.14	591.57	479.20
503.27	563.13	594.34	483.18
505.29	565.15	597.22	485.19
506.26	567.15	599.53	487.18
507.29	569.13	601.55	489.19
508.48	573.08	603.54	490.19
509.24	579.14	604.91	492.18
510.47	581.09	605.58	493.19
511.26	583.14	610.61	495.19
513.22	585.17	612.52	496.20
515.26	587.15	616.19	497.25
517.26	589.13	617.21	499.19
518.43	591.16	620.32	500.35
520.45	593.12	622.38	503.23
522.47	595.14	625.53	505.18
525.26	597.23	627.55	507.18
526.56	608.36	630.63	508.90
527.26	611.10	633.59	513.17
530.26	613.12	636.41	514.95
532.53	615.15	638.62	517.16
533.21	617.43	640.58	519.17
537.24	621.11	644.41	520.99
539.23	623.11	645.20	523.18
541.25	627.15	646.21	524.36
543.23	629.13	647.18	526.36
544.23	631.15	648.42	528.38
547.27	633.11	650.01	531.16
548.48	635.17	650.73	533.15
549.24	637.12	653.56	535.18
550.73	639.48	654.56	536.17
551.27	646.40	655.56	537.20
553.26	651.69	656.57	538.20
555.23	652.40	657.61	539.20
557.29	653.31	659.61	541.19
559.26	657.47	660.42	543.18
561.25	660.06	661.67	545.16
563.31	663.14	662.44	547.16
565.05	664.44	663.44	549.15
567.25	669.16	664.72	551.15
568.27	672.40	666.44	553.11
569.27	673.45	668.59	555.16
570.44	679.09	672.41	557.18
571.27	683.18	674.43	559.18
572.28	686.10	675.54	561.17
573.29	687.49	676.45	563.16
575.27	692.45	677.75	565.14
577.59	695.17	678.46	567.16

OO (+) m/z (406)	OO(-) m/z (294)	CC (+) m/z (324)	CC (-) m/z (374)
579.27	699.06	679.50	568.15
581.26	701.09	680.62	569.17
583.26	702.50	682.65	571.20
585.23	703.11	685.19	573.18
587.25	705.15	686.42	575.18
589.27	713.10	687.17	577.15
591.29	715.55	688.45	579.14
593.31	716.52	689.55	581.14
594.47	718.52	692.54	583.18
595.39	719.75	694.62	585.21
597.30	721.53	696.62	586.17
599.25	725.13	701.57	587.16
601.24	729.09	703.58	589.16
603.59	730.54	706.54	591.15
605.62	738.51	711.51	593.15
607.25	739.12	713.30	595.16
609.28	740.49	714.43	597.17
610.48	742.51	715.19	599.37
613.27	743.51	716.52	601.16
615.26	744.53	717.58	603.19
617.25	745.56	718.55	605.25
619.24	747.51	720.56	606.17
620.29	748.49	722.54	609.19
621.28	750.56	725.53	611.14
622.49	752.10	728.50	612.13
625.28	753.13	730.54	613.17
626.34	759.12	731.60	615.20
627.59	760.47	732.55	616.49
628.43	762.51	734.57	619.31
631.25	764.50	739.54	621.17
633.26	766.51	740.53	623.16
635.25	773.55	741.53	625.14
637.43	776.58	742.53	627.16
638.67	786.51	744.56	629.16
640.65	788.02	746.58	631.17
643.28	788.53	748.59	635.15
645.27	792.56	750.57	636.14
647.25	797.64	752.49	637.14
650.48	799.63	753.56	639.11
655.38	809.59	754.51	641.16
657.19	810.52	756.56	643.18
659.35	811.14	758.57	645.18
661.24	813.12	760.58	646.15
663.25	815.55	766.53	647.13
664.50	818.54	768.54	649.13
665.60	824.59	770.56	650.07
666.48	831.18	772.57	653.32
668.65	832.96	774.57	659.16
671.24	834.98	778.53	660.12
672.46	836.52	779.48	662.14
673.28	836.99	780.51	663.20
675.60	838.00	782.54	664.42
677.15	838.52	784.56	666.06
678.52	841.11	786.58	669.12
681.27	843.44	788.60	671.18
683.22	844.41	792.56	673.50
685.22	845.72	794.55	675.18
687.19	846.46	796.55	679.16
688.47	848.03	798.53	681.14
689.24	851.10	801.66	683.19
691.26	853.48	804.51	685.20
692.59	857.54	806.54	687.55
694.52	859.49	808.55	693.13
695.77	861.49	810.57	695.34
696.69	862.99	813.67	697.37
699.28	866.15	817.67	699.50
700.20	867.16	820.56	700.52
701.60	870.50	822.56	701.54
702.67	871.48	824.54	706.16
703.62	872.43	826.55	709.14
706.57	877.09	828.56	711.17
711.23	879.19	830.54	714.51
713.43	883.53	832.56	715.54
715.24	885.56	834.58	716.54
716.06	887.54	836.59	720.15
717.61	890.53	838.61	721.14
718.58	893.11	842.65	726.57
719.24	898.57	844.66	730.56
720.59	899.58	846.49	732.56
723.21	901.10	848.50	733.14
724.64	903.07	851.60	734.54
725.58	905.12	852.64	736.13
728.54	907.14	853.68	738.49
730.55	908.57	854.52	740.53
731.62	910.57	856.54	742.55
732.57	925.21	858.55	744.56
734.58	928.12	860.53	747.52
738.58	936.54	862.59	750.55
739.55	940.48	864.63	755.16
740.56	977.27	872.57	757.16
741.55		873.59	758.51
742.56		875.39	760.51
744.58		877.52	764.52
745.59		879.69	766.54
746.59		881.73	768.55
748.60		893.52	770.59
750.59		899.66	773.54
751.20		901.25	774.55
752.54		905.72	776.55
753.54		907.49	780.54
754.55		909.51	782.50
756.55		912.59	784.54

OO (+) m/z (406)	OO(-) m/z (294)	CC (+) m/z (324)	CC (-) m/z (374)
758.57		916.19	786.53
760.59		918.54	788.56
765.21		924.49	792.55
766.56		925.47	794.56
768.58		931.49	797.63
770.57		932.48	802.53
772.58		934.55	804.58
774.60		936.57	806.56
779.53		938.59	808.49
780.55		947.43	810.52
782.56		948.46	812.55
786.60		950.51	814.59
788.62		956.56	815.56
792.57		963.28	818.53
794.59		966.52	820.57
796.59		972.41	822.54
798.54		976.54	824.52
802.51		983.15	826.55
804.54			828.54
806.55			829.45
808.57			831.17
810.58			832.50
813.67			833.50
815.71			834.51
819.23			836.55
822.47			838.57
823.48			842.61
828.51			843.58
830.55			844.52
832.55			846.54
833.60			850.56
834.57			852.58
835.63			853.57
836.61			855.54
837.65			857.52
843.21			859.54
845.26			861.55
847.25			867.16
848.51			869.54
851.31			870.53
854.61			871.54
855.29			872.55
857.24			877.15
859.22			879.23
861.26			881.51
863.65			883.53
865.24			885.55
867.22			894.55
869.36			895.56
871.29			896.55
873.26			897.57
875.21			898.56
877.28			899.58
879.25			907.55
881.25			908.58
883.27			909.55
884.57			910.60
887.20			911.57
889.27			918.56
891.26			920.56
893.53			922.59
899.28			923.59
904.50			925.67
905.25			931.14
907.20			934.60
909.50			936.62
911.56			938.64
915.21			963.70
920.30			
922.50			
924.49			
926.51			
929.20			
932.55			
936.56			
938.48			
940.46			
943.43			
945.42			
948.50			
950.52			
954.51			
956.53			
958.53			
961.17			
962.44			
964.47			
965.47			
966.48			
971.20			
972.48			
974.50			
978.52			
981.26			
987.27			
989.41			
992.51			
994.62			
997.43			
999.49			
