# Supplementary material for: Docosahexaenoic acid mechanisms of action on the bovine oocyte-cumulus complex
Source: J Ovarian Res. 2017 Nov 9;10:74. doi: 10.1186/s13048-017-0370-z (PMC5679375; doi:10.1186/s13048-017-0370-z)
Supplement: Supplementary file 4 — Differential analysis of lipids in control and DHA 1 μM –treated oocytes and cumulus cells. (PDF 1215 kb) [file 13048_2017_370_MOESM4_ESM.pdf]

Additional file 4: Table S4: Differential analysis of lipids in control and DHA 1µM –treated oocytes and cumulus cells.

Oocytes (n=15)

m/z (mode)	*P-value*	*Normized peak height (mean ± SEM)*		*Annotation*
		**Control**	**DHA 1µM**	
387.24(+)	*0,0006*	1748.8±199.9	921.5±75.7	
518.42(+)	*0,0037*	1239.4±125.2	781.7±71.6	LPC 16:0 + Na^+
725.57(+)	*0,0065*	4455±416.1	2518.5±509.7	SM 34:1 + Na^+
965.47(+)	*0,0005*	162.6±14.1	253.8±18.3	

Cumulus cells (n=4)

m/z (mode)	*P-value*	*Normized peak height (mean ± SEM)*		*Annotation*
		**Control**	**DHA 1µM**	
625.53 (+)	*0,0036*	349.5 ± 16.6	221.7 ± 21.9	
630.63 (+)	*0,0014*	467.9 ± 31.4	242.5 ± 25.7	
758.57 (+)	*0,0096*	6351.4 ± 792.6	3164.1 ± 313.9	PC 34:2 + H^+
784.56 (+)	*0,0051*	3901.5 ± 393.2	2058.1 ± 168.9	PC 34:0 + Na^+
786.58 (+)	*0,0076*	9319.3 ± 1168.1	4279.2 ± 518.5	PC 36:2 + H^+
836.59 (+)	*0,0080*	1321.7 ± 137.5	756.9 ± 46.1	PC 38:2 + Na^+
838.61 (+)	*0,0085*	586.3 ± 43.6	387 ± 28.1	
