# Supplementary material for: Docosahexaenoic acid mechanisms of action on the bovine oocyte-cumulus complex
Source: J Ovarian Res. 2017 Nov 9;10:74. doi: 10.1186/s13048-017-0370-z (PMC5679375; doi:10.1186/s13048-017-0370-z)
Supplement: Supplementary file 5 — Possible annotation of differential m/z peaks. (PDF 224 kb) [file 13048_2017_370_MOESM5_ESM.pdf]

Additional file 5: Table S5. Possible annotation of differential m/z peaks

Imput m/z	Exact m/z	delta ppm	Ion	category	main class	sub class	name	Formula	Annotation
387.248	387.1802	0.0676	[M+H]+	FA	Fatty Acids an	Unsaturated fatty acids [FA0103]		C22H27O6	C 22:4
518.427	518.3217	0.10542	[M+Na]+	GP	Glycerophosp	Monoacylglyc	1-Palmitoyl-2-hydroxy-PC	C24H50NO7PNa	LPC(16:0)
713.426	713.4000	0.0265	[M+K+]	GP	Glycerophosp	Diacylglycerophosphocholines [GP0101]			PC(32:0)
725.577	725.5568	0.0205	[M+Na]+	SP	Phosphosphin	Ceramide phosphocholines (sphingomyelin		C39H79N2O6PNa	SM(d34:1)
758.568	758.5695	-0.0011	M+ H+	GP	Glycerophosp	Diacylglycerophosphocholines [GP0101]		C42H81NO8P	PC(34:2)
784.557	784.5827	-0.0260	M+ Na+	GP	Glycerophosp	Diacylglycerophosphocholines [GP0101]		C42H84NO8PNa	PC(34:0)
786.578	786.6008	-0.0225	[M+H]+	GP	Glycerophosp	Diacylglycerophosphocholines [GP0101]		C44H85NO8P	PC(36:2)
836.589	836.5412	0.0479	M+ Na+	GP	Glycerophosp	Diacylglycerophosphocholines [GP0101]		C46H88NO8PNa	PC 38:2
